# Genome Sequence of *Fusobacterium nucleatum* Subspecies *Polymorphum* — a Genetically Tractable Fusobacterium

**DOI:** 10.1371/journal.pone.0000659

**Published:** 2007-08-01

**Authors:** Sandor E. Karpathy, Xiang Qin, Jason Gioia, Huaiyang Jiang, Yamei Liu, Joseph F. Petrosino, Shailaja Yerrapragada, George E. Fox, Susan Kinder Haake, George M. Weinstock, Sarah K. Highlander

**Affiliations:** 1 Human Genome Sequencing Center, Baylor College of Medicine, Houston, Texas, United States of America; 2 Department of Microbiology and Molecular Genetics, University of Texas Medical School, Houston, Texas, United States of America; 3 Department of Molecular Virology and Microbiology, Baylor College of Medicine, Houston, Texas, United States of America; 4 Department of Biology and Biochemistry, University of Houston, Houston, Texas, United States of America; 5 Associated Clinical Specialties, University of California at Los Angeles School of Dentistry, Los Angeles, California, United States of America; Pasteur Institute, France

## Abstract

*Fusobacterium nucleatum* is a prominent member of the oral microbiota and is a common cause of human infection. *F. nucleatum* includes five subspecies: *polymorphum*, *nucleatum*, *vincentii*, *fusiforme*, and *animalis*. *F. nucleatum* subsp. *polymorphum* ATCC 10953 has been well characterized phenotypically and, in contrast to previously sequenced strains, is amenable to gene transfer. We sequenced and annotated the 2,429,698 bp genome of *F. nucleatum* subsp. *polymorphum* ATCC 10953. Plasmid pFN3 from the strain was also sequenced and analyzed. When compared to the other two available fusobacterial genomes (*F. nucleatum* subsp. *nucleatum*, and *F. nucleatum* subsp. *vincentii*) 627 open reading frames unique to *F. nucleatum* subsp. *polymorphum* ATCC 10953 were identified. A large percentage of these mapped within one of 28 regions or islands containing five or more genes. Seventeen percent of the clustered proteins that demonstrated similarity were most similar to proteins from the clostridia, with others being most similar to proteins from other gram-positive organisms such as *Bacillus* and *Streptococcus*. A ten kilobase region homologous to the *Salmonella typhimurium* propanediol utilization locus was identified, as was a prophage and integrated conjugal plasmid. The genome contains five composite ribozyme/transposons, similar to the Cd*ISt* IStrons described in *Clostridium difficile*. IStrons are not present in the other fusobacterial genomes. These findings indicate that *F. nucleatum* subsp. *polymorphum* is proficient at horizontal gene transfer and that exchange with the *Firmicutes*, particularly the *Clostridia*, is common.

## Introduction

*Fusobacterium nucleatum* is a Gram-negative anaerobic species of the phylum *Fusobacteria*, numerically dominant in dental plaque biofilms, and important in biofilm ecology and human infectious diseases. Dental plaque is a complex and dynamic microbial community that forms as a biofilm on teeth, and harbors more that 400 distinct species *in vivo* [1]. *F. nucleatum* is a prominent component quantitatively and is one of the first Gram-negative species to become established in plaque biofilms [2], [3]. It is a central species in physical interactions between Gram-positive and Gram-negative species [4] that are likely to be important in biofilm colonization, and contributes to the reducing conditions necessary for the emergence of oxygen-intolerant anaerobes [5]. *F. nucleatum* is also one of a small number of oral species that is consistently associated with, and increased in number at, sites of periodontitis [1], [2], [6], one of the most common infections of humans [7]. It is one of the most common oral species isolated from extraoral infections, including blood, brain, chest, lung, liver, joint, abdominal and obstetrical and gynecological infections and abscesses [6], [8]–[14]. Further, *F. nucleatum* is a common anaerobic isolate from intrauterine infections and has been associated with pregnancy complications including the delivery of premature low birth weight infants [13], [15]–[19]. Thus, *F. nucleatum* is a significant pathogen in human infections, including several infections with significant societal impact.

There are five recognized subspecies of *F. nucleatum*: *polymorphum*, *nucleatum*, *vincentii*, *fusiforme*, and *animalis*. DNA sequence analysis of the genome of *F. nucleatum* subspecies *nucleatum* (ATCC 25586) (FNN) and *vincentii* (ATCC 49256) (FNV) indicate clear differences in genetic content between the strains. Three hundred forty-six 346 (17%) and 441 (20%) of the ATCC 25586 and ATCC 49256 open reading frames (ORFs) are absent from the other strain, respectively, and rearrangements are evident in the ORFs present in both strains [20], [21]. Phenotypic heterogeneity among *F. nucleatum* strains has led to the concept that it is a “species complex” [22], [23]. Taxonomic studies confirm that *F. nucleatum* ssp. *polymorphum* ATCC 10953 (FNP) represents a phylogenetic branch separate from the previously sequenced *F. nucleatum* strains. This branch includes significant human pathogens [12], [24], [25]. Phenotypic investigations of ATCC 10953 have characterized its uptake and metabolism of amino acids, simple sugars and peptides [26]–[29], its physical interaction with epithelial, connective tissue and host immune cells [30]–[32], its ability to modulate host immune cell function including the induction of apoptosis [30], [33]–[35], its ability to enhance the survival of strict anaerobes in biofilm and planktonic multispecies cultures [5], [36], and its ability act synergistically with other oral pathogens to enhance virulence in animal model systems [37], [38]. In addition, ATCC 10953 harbors a single native plasmid, pFN3 [39], and has been shown to be amenable to gene transfer [40].

The distinct taxonomic status, relatively extensive phenotypic analyses, and genetic transformability of *F. nucleatum* ssp *polymorphum* ATCC 10953 suggested that genomic analysis would prove valuable to subsequent studies of this species. Thus, we determined, analyzed, and present the genomic DNA sequence of *F. nucleatum* subspecies *polymorphum* strain ATCC 10953. Analysis of the FNP genome revealed that 25% of the genes identified are not represented in the previously sequenced fusobacterial genomes, and that horizontal gene transfer (HGT) has contributed to the evolution of this strain.

## Results and Discussion

### Genome anatomy

**Figure 1:**
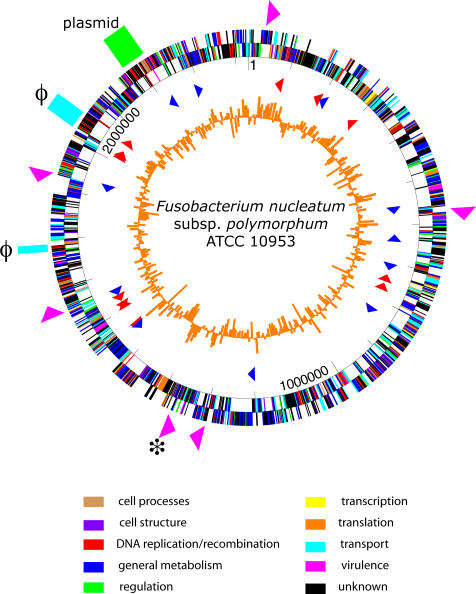
Map of the FNP ATCC 10953 genome. The inner circle (orange) shows the percent GC calculated using a sliding window of 5 kb. The triangles in the next circle show the location and directionality of tRNAs (red) and ncRNAs (blue). The next tract shows the coordinate scale; this is surrounded by the ORFs, on both strands. ORFs are colored by category, as follows: tan, cell processes; purple, cell structure; red; DNA replication and recombination; blue, general metabolism; green, regulation; yellow, transcription; orange, translation, cyan, transport; fuchsia, virulence; and black, unknown. The IStrons are indicated by the fuchsia arrowheads on the outside circle; the intact IStron is indicated with the star. Plasmid (green) and phage locations (cyan) also appear on the outside circle.

**Figure 2:**
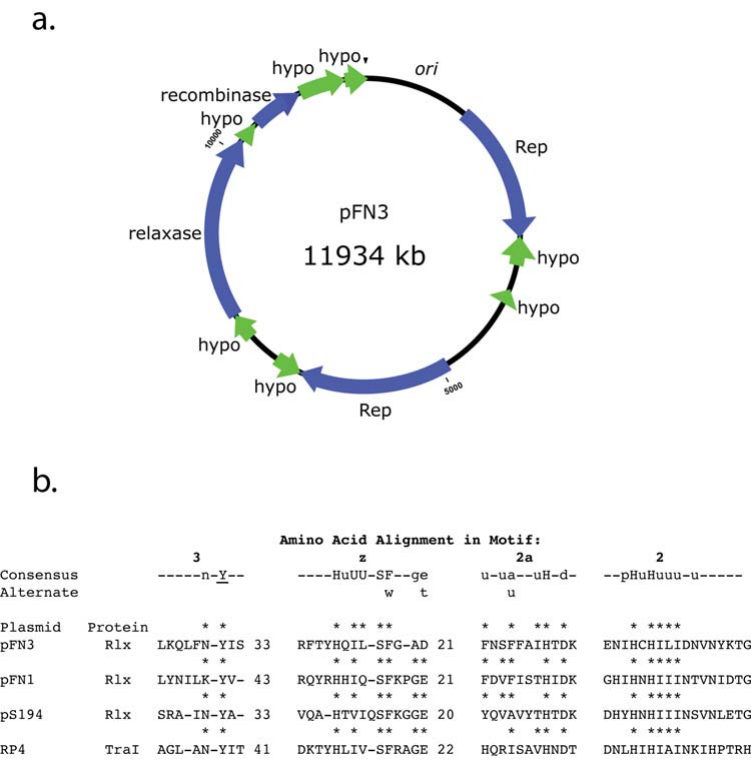
Plasmid pFN3. a) Map of plasmid pFN3. Replication and recombination ORFs are shown in blue and hypotheticals are colored green. b) Alignments of fusobacterial relaxase protein domains to mobilization class consensus motifs [66]. Consensus sequence abbreviations: uppercase letters, conserved; lowercase letters, present in 50% of sites; U or u, bulky hydrophobic residues (I, L, V, M, F, Y and W); -, no consensus at this site; Y, putative active site tyrosine residue. Asterisks (*) above residues indicate identity with consensus sequence. Alignments were performed using Clustal W [102] and then adjusted to best fit the consensus.

The FNP genome consists of a single circular chromosome containing 2,429,698 bp (Figure 1, accession number CM000440) and a single circular plasmid of 11,934 bp (Figure 2, accession number CP111710). The FNP genome is larger than the FNN genome (2,174,499 bp), and the unfinished FNV genome (2,118,259 bp). The GC content of the chromosome is 26.84%, similar to FNN and FNV (Table 1) and the GC content for the plasmid is 24.53%. There are 2,433 predicted ORFs, 42 pseudogenes, 45 tRNA, 15 rRNA, and 11 ncRNA genes in the FNP genome.

**Table 1:** General genome statistics for FNP, FNN and FNV.

Genome	Length (bp)	%GC content	% Coding	ORFs	Proteins	rRNA	tRNA	ncRNA
FNP^a^	2,429,698	26.84	94.28	2510	2391	ND	45	15
FNN^b^	2,174,499	27.15	89.10	2129	2067	15	47	ND
FNV^c^	2,118,259	27.56	ND	2277	>2212	12	43	ND

a *F. nucleatum* subsp. *polymorphum* ATCC 10953, this project, accession number CM000440.

b *F. nucleatum* subsp. *nucleatum* ATCC 25586, accession number NC_003454.

c *F. nucleatum subsp. vincentii* ATCC 49256, accession number NZ_AABF00000000.

ND, not determined.

Forty-two of the tRNAS lie in one of seven clusters, each containing from two to fourteen tRNAs. Identical clusters, in both content and internal gene order, are found in FNN. However, the relative position of the clusters in the genome is different. In addition, FNN has two additional asparagine tRNAs, which are associated with rRNA gene regions that have not been fully sequenced in ATCC 1095300 so it is possible that these tRNAs are present in FNP. The tRNAs represent all twenty amino acids. In five cases (Ala, Cys, His, Trp, Tyr), there is only a single tRNA. Many of the tRNAs are duplicated (Asn, Gln, Gly, Leu, Pro, Lys, Ser, Thr, Val) or triplicated (Asp, Glu). The clusters include duplicates as well as singleton tRNAs with no clear pattern associated with the distribution of singletons, duplicates and triplicates. Some tRNAs have multiple copies only one of which is unique, e.g. Gly, Lys, Ser, Val. All four Arg tRNAs, the three Met tRNAs (one is identified as the likely initiator) and the two Phe tRNAs have unique sequences.

**Figure 3:**
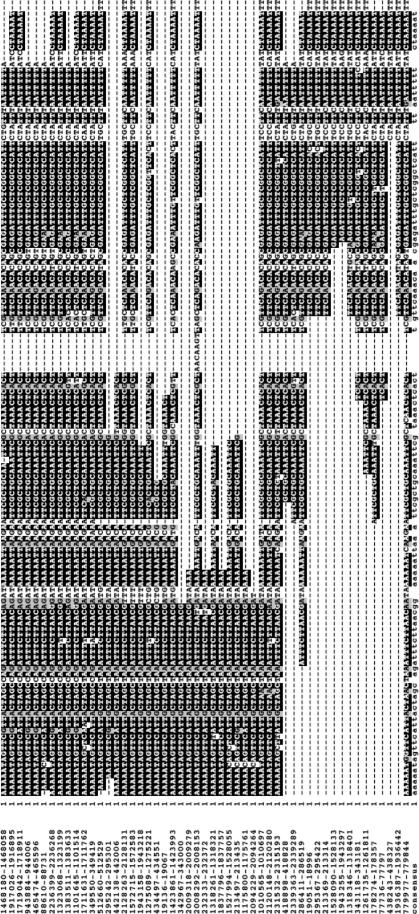
Intergenic repeats. The repeats were aligned using ClustalW [102] and conserved bases were shaded using BOXSHADE (www.ch.embnet.org/software/BOX_form.html). Coordinates are shown in the left column.

A 131 bp repeat sequence occurs seventeen times within the genome (Figure 3). In several cases, the repeat regions have the potential to encode small hypothetical peptides, which we believe were incorrectly annotated as ORFs in FNN and FNV. All of the 131 nt. repeats from FNP were aligned and used to generate a consensus sequence for additional BLASTN searches. Five nearly complete repeats (≥90% of the element's length) and eight repeats with gaps were observed. Sequences corresponding to the 3′ half of the element were also found in six locations. In most cases, the repeat sequence was found in intergenic regions and not within coding sequence. One complete copy of the repeat and numerous subsequences were identified in FNN and twenty-eight complete copies plus numerous subsequences were identified in FNV. As in FNP, these fell within intergenic regions. Because the long repeats occur within intergenic regions, it is possible that the sequence is involved in gene regulation, though no particular regulatory motifs were found within the sequence.

Many examples of conservation of gene order, operon structure and gene clustering were observed in the genome. Most of the ribosomal protein genes are organized into operons similar to the L11, L10, *str*, S10, *spc* and α operons in *Escherichia coli*; each encodes between two and eleven ribosomal proteins [41]. Several non-r-protein clusters, similar to conserved non-r-protein gene clusters described in *E.coli* and other bacteria [42] were also identified in the FNP genome. Table 2 shows the conservation of these gene regions between FNP, FN, *Clostridium difficile* (NC_009089), *Bacillus anthracis str. Ames* (NC_003997), *E.coli* MG1655 (NC_000913). The conserved gene clusters were categorized in five groups according to protein function. Clusters 1–11 in group I are primarily RNA and protein constituents of the ribosome. Group II encodes subunits of the F-type two-sector ATPase. Proteins encoded by the group III clusters are involved in RNA synthesis, modification, transcription, and translation. Cluster 17 in group IV, encodes a spermidine/putrescine ATP binding cassette (ABC) superfamily transporter, while group V contains cluster 18, which codes for the molecular chaperones GroEL and GroES.

**Table 2:** Conserved gene clusters/operons in FNP.

Group	Cluster	Conserved gene cluster	FNN	*C. difficile*	*B. anthracis*	*E. coli*
I	1	16S, 23S, 5S rRNAs	ND	+	+	+
	1	S10 operon (*rpsJ, rplC, rplD, rplW, rplB, rpsS, rplV, rpsC, rplP, rpmC, rpsQ*)		+	+	+
	2	Str operon (*rpsL, rpsG, fusA*)	+	+	+	+
	4	Spc operon (*rplN, rplX, rplE, rpsN, rpsH, rplF, rplR, rpsE, rpmD, rplO, secY*)	-a	+	+	+
	5	L13 operon (*rplM, rpsI*)	+	+	+	+
	6	L11 operon (*rplK, rplA*)	+	+	+	+
	7	Alpha operon (*rpsM, rpsK, rpoA, rplQ*)	+	+	+	+
	7	L35 operon (*infC, rpmI, rplT*)	+	+	+	+
	8	No L34 operon	+	-b	-b	-b
	10	L21/L27 (*rplU, rpmA*)	+	+	+	+
	11	L10 operon (*rplJ, rplL*)	+	+	+	+
II	12	ATPases (*atpB, atpE, atpF, atpH, atpA, atpG, atpD, atpC*)	+	+	+	+
III	13	Beta operon (*rpoB, rpoC*)	+	+	+	+
	14	Initiation factor cluster (*nusA*, ABC1 family protein, *infB*)	+	-c ^,^ d	-c ^,^ d	-c
	15	RF-1 cluster (*prfA, hemK*)	+	+	+	+
	16	Ribosome release factor cluster (*rpsB, tsf, pyrH, rrf*)	+	+	+	+
IV	17	Spermidine/putrescine ABC transport cluster (*potA, potB, potC, potD*)	+	+	+	+
V	18	Chaperones cluster (*groES*, *groEL*)	+	+	+	+

a The *rplX* gene is duplicated in this operon in the FNN genome.

b The L34 operon is present in the *C. difficile, B. anthracis* and E. *coli* genomes.

c The ABC1 family protein gene is missing.

d An L8A gene maps between *nusA* and *infB.*

ND, not determined.

### Genome Comparisons

Slightly more than 62% of the FNP genes (1514) are found in both of the previously sequenced fusobacterial genomes. Thus, nearly 38% of the genome is either wholly unique to FNP or is shared by FNP and only one of the other two genomes. In terms of coding potential, these 919 genes account for the differences between FNP, FNN, and FNV and thus serve to distinguish FNP. Three comparative lists have been generated: ORFs unique to FNP with respect to FNN and FNV; ORFs common to FNP and FNV, but absent in FNN; and ORFs common to FNP and FNN, but absent in FNV (Table S1).

627 FNP ORFs have no ortholog in either FNN or FNV (Table S1a), including 106 conserved hypothetical proteins, 287 hypothetical proteins and 9 pseudogenes. Thirty-eight ORFs functioning in transport, including transporters of amino acids, oligopeptides, a siderophore, and divalent metals (Hg^2+^, Cu^2+^, Ni^2+^) are also unique. Seven additional membrane proteins, two phosphotransferases, and two beta-lactamases are also present only in FNP. Twenty-seven ORFs related to transcriptional regulation are unique to FNP, including a LuxS autoinducer ortholog and two sensor histidine kinases. Additionally, FNP encodes several unique proteins related to DNA modification. These include such functions as methylation, histone acetylation, recombination, integration, topoisomerase, and type I restriction and modification. FNP also contains numerous prophage and transpose genes not found in FNN or FNV. Four Tra conjugation genes (FNP_1868–1871) were found only in FNP. These are adjacent to a region encoding two proteins resembling Type IV secretion components, an outer membrane protein, and four hypothetical proteins. Thus, it is plausible that FNP may have obtained a Type IV secretion system via HGT. This region and the prophage sequences are discussed in more detail, below.

Ninety-six ORFs (5 are pseudogenes) are shared between FNP and FNV but are missing from FNN (Table S1b), including 20 conserved hypothetical proteins and 36 fusobacterial conserved hypothetical proteins. Citrate lyase, glutamate–ammonia lyase, serine-pyruvate aminotransferase, cholinephosphate cytidylyltransferase, sulfate reductase and malate dehydrogenase enzymes are shared by FNV and FNP. They also each contain two N-acetylmuramoyl-L-alanine amidases not found in FNN. Five regulatory proteins are common to FNP and FNV, including transcriptional regulators of the MarR, MerR, LysR, and Crp families, and the RNA polymerase sigma factor σ^54^.

Two hundred thirteen ORFs (5 are pseudogenes) are found in both FNP and FNN but not in FNV (Table S1c). The total may be inflated, however, because the FNV genome is incomplete and some ORFs may have been missed. This set includes 35 conserved hypothetical proteins and 49 fusobacterial conserved hypothetical proteins. There are 30 transport proteins in this group, including iron transporters, a siderophore transporter, amino acid symporters, ion symporters, and a formate/nitrate transporter. Twelve ORFs related to genetic regulation are in this group, including RpiR and TetR family regulators, Fur, an iron-dependent transcriptional regulator and a response regulator and sensor histidine kinase for the ethanolamine utilization pathway. Also common to FNP and FNN are the GroeSL chaperonins, a cold shock protein, a ribosome-related heat shock protein, and a translation inhibitor. Other proteins of interest in this group are a beta-lactamase, bacterioferrin, at least 12 genes encoding the ethanolamine utilization gene family, an autotransporter/adhesin, an O-antigen assembly gene, 5 glycosyltransferases and the gene for a possible immunosuppressive protein FipA [43], [44].

### Horizontal Gene Transfer

HGT can be detected by several parametric methods based on deviant nucleotide composition [45], [46], dinucleotide frequencies [47], codon usage biases [48]–[50] or patterns inferred by Markov chain analysis [51]. Phylogenetic methods determine a gene's unusual similarity or distribution among organisms by comparing phylogenetic trees of different genes from the genome and assessing the significance of any resulting incongruities. Alternative phylogenetic methods exist that do not reconstruct phylogenetic trees like Clarke's phylogenetic discordance test [52] and Lawrence's [53] rank correlation test. Another reliable inference of recent HGT events is the anomalous phylogenetic distribution method wherein a gene is present in one genome but not found in several closely related genomes [54]. This is the approach used examine genes that had no top BLAST hit to either of the two sequenced fusobacterial genomes, FNN and FNV.

**Figure 4:**
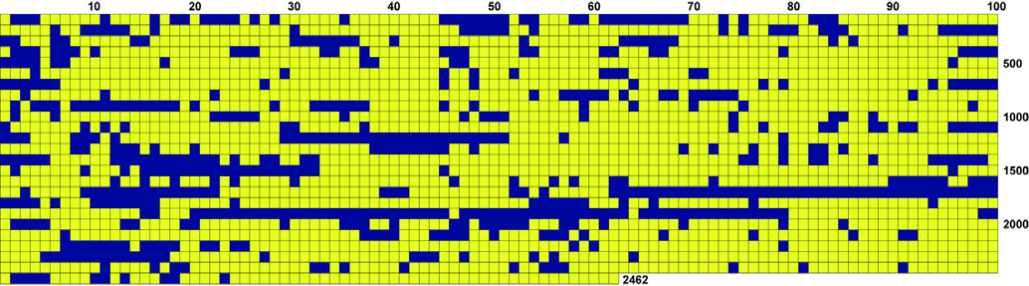
Whole genome display of FNP illustrating clustering of genes without hits in FNN or FNV. Yellow boxes represent FNP genes with either FNN or FNV as top BLASTN hits (1835/2462 or 75%) and blue boxes represent genes whose top BLASTN hits are to genes from other organisms (627/2462 or 25%).

Based on BLASTP similarity searches, a total of 1235 ORFs, composed of the 621 FNP ORFs and 9 pseudogenes with no top hits to FNN or FNV (Table S1a) and 608 hypothetical or conserved hypothetical proteins, were graphically plotted to identify clusters that could represent regions of HGT (Figure 4). About 21% of these, or 255 ORFs, mapped within gene clusters. There were 28 specific regions or islands of interest with clusters of 5 or more genes. Top BLASTP hits for each cluster (Table S2) were examined to determine a consensus genus and species.

One hundred forty of the ORFs (out of 255), or 55% were hypothetical proteins with no matches to other bacterial proteins. Of the remaining 115, 20 ORFs or 17% had top hits to the *Clostridia*. The most common top hits in this class were to *Clostridium tetani*, *Clostridium thermocellum, Clostridium perfringens,* and *Desulfitobacterium hafniense*. Other ORFs had top hits to other *Firmicutes* including *Bacillus*, *Streptococcus, Listeria* and *Enterococcus* species. Hits to the archaea *Methanosarcina mazei, Methanococcoides burtonii,* and *Methanothermobacter* species, as well as to cyanobacteria *Nostoc punctiforme*, *Trichodesmium erythraeum* and *Synechocystis* sp., were also observed.

A 10 kilobase (kb) region of the FNP genome from nt. 27349 to 37954 (FNP_2111–FNP_2124) appears to have arisen via HGT since this region is not found in the other published fusobacterial genomes and since its GC content (30.4%) is higher than that of the remainder of the genome (26.8%). In the *Firmicutes*, the clostridial %GC ranges from 28.5 to 30.9 so the DNA may have been acquired from this genus. The region includes 14 predicted genes, 5 of which compose Cluster I (Table S2). Twelve of the genes have no orthologs in FNN or FNV. The genes in this region are homologous to the propanediol utilization locus (*pdu*) of *Salmonella enterica* serovar typhimurium, which also arose via HGT [55]. Propanediol is a byproduct produced during the fermentation of fucose [55], [56], which has been shown to be present in saliva and metabolized by oral bacteria [57]. Although it appears that FNP is missing the fucose catabolism (*fuc*) operon, some bacteria such as *E. coli* secrete propanediol [58], so it is possible that FNP can utilize this propanediol pool.

According to Kapatral *et al.*, the genomes of FNN and FNV lack the necessary enzymes for valine, isoleucine, and leucine biosynthesis [20], [21]. A region in the FNP genome carries the *ilv*/*leu* operon, which is responsible for the biosynthesis of these amino acids. The predicted products encoded by this locus include dihydroxy-acid dehydratase (IlvD, FNP_0059), threonine ammonia-lyase (IlvA, FNP_0060), acetolactate synthase (IlvB and IlvN, FNP_0061 and FNP_0062), 2-isopropyl malate synthase (LeuA, FNP_0063), 3-isopropyl malate synthase (LeuC and LeuD, FNP_0064 and FNP_0065), isopropyl malate dehydrogenase (LeuB, FNP_0067), and ketol-acid reductoisomerase (IlvC, FNP_0069). The cluster of unique genes (Cluster IV) also includes a small hypothetical protein gene between *leuD* and *leuB* (FNP_0066). With the exception of *ilvA*, all of these genes are missing from the genomes of the other two sequenced fusobacteria. Three additional *ilv* genes are located at non-adjacent loci in the genome including an additional copy of *ilvA* (FNP_1302), which is not in the other genomes, and two copies of *ilvE* (branched-chain-amino-acid transaminase), one that is unique to *F. nucleatum* ATCC 10953 (FNP_1952) and one that is also found in the other two genomes (FNP_1165).

**Figure 5:**
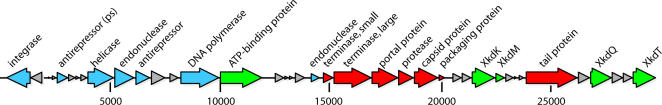
Linear map of prophage located between nts 2,024,189 and 2,053,649 in FNP. Replication and regulatory ORFs are colored blue, ORFs encoding structural proteins are red, ORFs encoding proteins with homologs in the nr database but of unknown function are colored green, and hypotheticals are shaded gray.

A prophage genome was identified immediately downstream of an arginine tRNA gene between coordinates 2024189 and 2053649 (28.9% GC) (Figures 1 and 5). The ORFs are not found in FNN or FNV (Table S1a). Forty-two open reading frames (FNP_1662–1703) were predicted in this region spanning four clusters of unique genes (XXI–XXIV), including genes encoding integrase, DNA polymerase, antirepressor, helicase, and terminase proteins. Several genes encoding bacteriophage structural components, such as capsid and tail proteins, were also identified in the region, though 20 of the open reading frames encode hypothetical proteins. The predicted tail proteins were most similar to those in a potential prophage genome in *C. tetani* E88 [59], while the terminase and packaging proteins were most similar to those in the *C. perfringens* phage phi3626 [60]. High scoring matches to the non-structural proteins were found in other gram-positive genomes, such as *Bacillus halodurans, Streptococcus mitis*, and *C. thermocellum*. Homologs of 10 of the phage proteins were found in FNV, though only one (a helicase, FNP_1671) was found in FNN. An additional block of phage-like genes map between coordinates 1775962 and 1786106 (FNP_1415–1432) (Figure 1 and Table S2, Cluster XVIII). Only one of the 19 proteins encoded in this region had orthologs in the other fusobacteria and only two of the proteins (a replication protein and integrase) matched to other bacteriophage sequences.

The region between nts. 2174023 and 2218775 in the FNP genome (FNP_1820–1879, Figure 1), containing 59 genes that include Clusters XXV–XXVII (25.4% GC), is predicted to contain a large conjugal plasmid (Figure 1, Table S1a, Table S2). Fifty-five unique genes, not found in FNN or FNP, encode a primase/helicase that could function as a replication initiation protein, topoisomerase, integrase, recombinase, a plasmid partitioning protein, and pseudogenes of a mobilization protein and a plasmid addiction system. This region also carries genes encoding homologs of seven Type IV secretion system (T4SS) proteins. T4SS can translocate DNA and proteins out of the bacterial cell to recipient cells; bacterial conjugation systems are a subset of this family [61]. Full-length copies genes encoding the T4SS proteins VirB4, VirB8, VirB10, VirB11 and VirD4 (FNP_1868–1871, 1873, 1875) are found within the conjugal plasmid region, as are truncated versions of VirB6 and VirB9. These proteins are most similar to orthologs in *Mesorhizobium*, *Ralstonia*, *Pseudomonas*, *Caulobacter* and *Rhodopseudomonas*. The T4SS proteins identified in FNP could constitute the inner membrane and periplasmic components of the transporter, but genes encoding components for biogenesis of the T-pilus (VirB1, VirB2, VirB3, VirB5 and VirB7) are missing. A different set of proposed Type IV pilus genes are present in FNP. A cluster of eleven genes (FNP_2389–2399) plus an unlinked *pilT* gene may encode the pilus, as suggested by Desvaux, et al. [62].

Five composite ribozyme/transposons, similar to the Cd*ISt* IStrons described in *C. difficile* [63] were identified in the genome (Figure 1). The consensus IStron in FNP is 1811 nt. long and contains a 477 nt. intron followed by an open reading frame encoding the transposase-like protein, TlpB. The FNP IStron is 31% identical to Cd*ISt*-C34 from *C. difficile* and contains four conserved RNA sequences that form the catalytic core of group I introns [64]. All five IStrons in FNP are inserted directly downstream of the pentanucleotide TTGAT, which is the conserved site of insertion of the IS*8301* family of transposons [65]. The IStrons have an average G+C content of 29%, consistent with that of both fusobacteria and clostridia. In *C. difficile,* Cd*ISt1* self-splices to remove itself from the mRNA into which it is inserted. As a result, the insertion does not disrupt expression of the gene. In FNP, we predict that only one copy of the IStron (1361160 to 1362978) is a fully functional element with self-splicing and transposition activities, since the other copies have mutations in either the ribozyme or *tlpB* regions of the element. Three additional sequences with homology to portions of the ribozyme were identified in the FNP genome and ten additional copies of *tlpB*-like genes occur in the genome. Homologs of this element were not found in any other organism, including the two strains of *Fusobacterium* that have been previously sequenced, though TlpB sequences are found in a variety of organisms, including cyanobacteria, *Bacillus cereus, Enterococcus, Deinococcus* and *Exiguobacterium*. Thus, it appears that a unique exchange between *C. difficile* and FNP has occurred.

ATCC 10953 harbors a single plasmid, pFN3 [40] (Figure 2a), which is 11,934 bp in length and has a GC content of 24.53%. Eleven pFN3 ORFs were identified: two possible replication protein genes, a possible resolvase/recombinase gene, a DNA relaxation protein gene and seven hypothetical protein genes. The two replication protein genes (FNP_pFN3g01 and FNP_pFNgo5) have predicted protein sequences with 20–22% identity and 27–32% similarity to the putative replication protein of the *F. nucleatum* native plasmid pFN1 [40]. The sequence upstream of the pFN3 replication protein gene at 1315 has a sequence (1007 to 1136) characterized by clusters of two overlapping 18 bp repeats (repeat 1: TAATAGTACAAATTTCCC; repeat 2: TAGTACAAATTTCCCGAT). Several of the repeats are spaced at 22 bp intervals, suggesting that they may represent replication protein binding sites that are characteristic of the replication origin of iteron-regulated plasmids. The resolvase (FNP_pFN3g09) was identified based on the presence of a N-terminal resolvase domain (pfam02796). The DNA relaxation protein (relaxase) (FNP_pFN3g07) has a relaxase domain and contains the conserved consensus motifs defined for relaxase proteins [66] (Figure 2b). The pFN3 resolvase and relaxase genes both have potential significance for HGT. Resolvases are important in DNA recombination events, including excision and integration of mobile DNA elements. Relaxase proteins mediate the initiation of conjugal transfer of plasmid DNA. Plasmids that encode relaxases, which are not conjugative themselves, may be mobilized with the additional conjugative functions provided *in trans*. Two other native *F. nucleatum* plasmids, pFN1 (AF159249) and pPA52 (AF022647), which are 98% identical, also carry relaxase genes [40]. The occurrence of the relaxase genes suggests the possibility that these plasmids were introduced into *F. nucleatum* by conjugative processes. Consistent with this mechanism of HGT is the finding that plasmids or DNA sequences related to pPA52 have been detected in 18% of *F. nucleatum* strains examined [67].

### Virulence

We identified 132 predicted proteins that may play a role in fusobacterial virulence (Table 3). Most of these are found in FNN and FNV, though there are a few notable exceptions. As in the two previously sequenced fusobacterial genomes [20], [21], we identified a VacJ homolog (FNP_0314). This protein has been shown to play a role in the intracellular spread of *Shigella flexneri* [68]. Although its mechanism of action has not been examined, VacJ may play a similar role in FNP since recent evidence suggests that *F. nucleatum* can invade epithelial cells [32], which may allow dissemination throughout the host to cause infections at non-oral sites [69]. Other previously known virulence factors were identified in FNP including the porin FomA (FNP_0972; not in FNV) [70], [71], MviN (FNP_1360), which plays a role in virulence in *Salmonella typhimurium* [72], TraT (FNP_1881) which provides resistance to complement [73], and VacB (FNP_1921), a ribonuclease involved in virulence gene expression in *S. flexneri* [74], [75]. The strain also carries genes for butyrate fermentation (FNP_0790, 0791, 0969, 0970, 0971, 1762 and either 1467 or 2146). The production of butyrate has been associated with mouth odor and gingival inflammation [76]. We also include FipA (described above) as a virulence factor because of its immunosuppressive properties [43], [77], though it is most similar to an acetyl-CoA transferase of the butyrate fermentation system. The *fipA* gene is not present in FNV.

**Table 3:** Potential virulence factor genes.

Locus_Tag	Start	Stop	Gene	Definition
*Miscellaneous virulence factors*				
FNP_0314*	693051	692284	*vacJ*	VacJ family lipoprotein
FNP_0972*	1330393	1329281	*fomA*	porin FomA
FNP_1118	1459567	1458752	*bacA*	undecaprenol kinase
FNP_1264	1619294	1618293		probable microcin C7 self-immunity protein MccF
FNP_1337	1692327	1693952	*fbpA*	fibronectin-binding protein A
FNP_1360	1721587	1723056		MviN family protein
FNP_1391	1750650	1752584		possible autotransporter adhesin
FNP_1446	1797366	1798136		probable Bvg family transcriptional regulator
FNP_1762*	2106322	2107530	*fipA*	acetyl-CoA acetyltransferase/immunosuppressive protein FipA
FNP_1880	2219936	2219178		von Willebrand factor domain protein
FNP_1881	2220088	2220807	*traT*	complement resistance protein TraT
FNP_1888	2226995	2228584		von Willebrand factor domain protein
FNP_1921	2263322	2265424	*vacB*	ribonuclease R
*Butyrate fermentation*				
FNP_2146	62092	60956		butyryl-CoA dehydrogenase
FNP_0790	1158356	1159132		3-hydroxybutyryl-CoA dehydratase
FNP_0791	1159148	1159987	*fadB*	3-hydroxybutyryl-CoA dehydrogenase
FNP_0969	1326816	1326151	*atoA*	butyrate-acetoacetate CoA-transferase, beta subunit
FNP_0970	1327487	1326834	*atoD*	butyrate–acetoacetate CoA-transferase, alpha subunit
FNP_0971	1329020	1327641	*atoE*	MFS superfamily major facilitator short chain fatty acids symporter
FNP_1467	1817668	1818813		butyryl-CoA dehydrogenase
FNP_1762*	2106322	2107530	*fipA*	acetyl-CoA acetyltransferase
*Iron acquisition*				
FNP_2267	200871	200098	*hmuV*	heme ATP binding cassette transporter, ABC protein
FNP_2268	201893	200868	*hmuU*	heme ATP binding cassette transporter, membrane protein HmuU
FNP_2269	202768	201896	*hmuT*	heme ATP binding cassette transporter, binding protein HmuT
FNP_2270	205012	203039		possible TonB-dependent iron (Fe) receptor
FNP_2353	300274	299846	*fur*	ferric uptake regulator protein
FNP_0006	386027	386275		possible alpha-hemolysin
FNP_0155*	520499	522103	*tpsB2*	probable TPS family two-partner secretion family protein TpsB
FNP_0156*	522114	530369	*tpsA2*	probable TPS family two-partner secretion family exoprotein TpsA
FNP_0159	531328	532326		possible hemolysin
FNP_0338	716299	716574		cobalamin/iron ATP binding cassette transporter, ABC protein
FNP_0339	716593	717216		cobalamin/iron ATP binding cassette transporter, ABC protein
FNP_0340	717349	718404		cobalamin/iron ATP binding cassette transporter, binding protein
FNP_0341	718423	720105		cobalamin/iron ATP binding cassette transporter, membrane protein
FNP_0428	795363	796424		iron ABC superfamily ATP binding cassette transporter, binding protein
FNP_0429	796439	797554		iron ABC superfamily ATP binding cassette transporter, ABC protein
FNP_0430	797544	799190		iron ABC superfamily ATP binding cassette transporter, membrane protein
FNP_0531	896606	895221		OfeT family oxidase-dependent iron transporter
FNP_0999	1350397	1351044		possible hemolysin III
FNP_1246*	1599267	1589866	*tpsA1*	probable TPS family two-partner secretion exoprotein TpsA
FNP_1247*	1601074	1599281	*tpsB1*	pseudogene of TPS family two-partner secretion protein TpsB
FNP_1451	1801085	1801975		iron ABC superfamily ATP binding cassette transporter, binding protein
FNP_1452	1802030	1804210		iron ABC superfamily ATP binding cassette transporter, binding protein
FNP_1453	1805190	1805966		iron ABC superfamily ATP binding cassette transporter, membrane protein
FNP_1454	1805980	1805966		iron ABC superfamily ATP binding cassette transporter, ABC protein
FNP_1660	2022933	2021746		probable Nramp family metal ion transporter
FNP_1765	2114622	2112373		possible TonB-dependent iron (Fe) receptor
*Drug transporters*				
FNP_0174	546224	547564		MOP/MATE family multidrug-resistance efflux pump
FNP_0388	760374	759283	*perM*	probable MFS superfamily major facilitator transporter, macrolide symporter
FNP_0507	872530	869465		RND superfamily resistance-nodulation-cell division antiporter
FNP_0508	873639	872533		RND superfamily resistance-nodulation-cell division antiporter
FNP_0622	986693	987907		probable MFS superfamily major facilitator transporter, multidrug symporter
FNP_0640	1009181	1010527	*norM1*	MOP/MATE superfamily multidrug-resistance efflux pump NorM
FNP_0769	1137188	1136280		DMT superfamily drug/metabolite transporter
FNP_0890	1260254	1261621	*norM2*	MOP/MATE family multidrug-resistance efflux pump NorM
FNP_1162	1513737	1512364	*norM3*	MOP/MATE family multidrug-resistance efflux pump NorM
FNP_1207	1557921	1559300		MOP/MATE family multidrug-resistance efflux pump
FNP_1299	1653389	1652052	*norM4*	MOP/MATE family multidrug-resistance efflux pump NorM
FNP_1503	1864175	1865314		possible MFP membrane fusion protein family transporter
FNP_1504	1865341	1866003		antimicrobial peptide ATP binding cassette transporter, ABC protein
FNP_1505	1866000	1867226		antimicrobial peptide ATP binding cassette transporter, membrane protein
FNP_1524	1881745	1882815		possible DMT superfamily drug/metabolite transporter
FNP_1596	1955589	1956911		MOP/MATE family multidrug-resistance efflux pump
*Beta-lactamases*				
FNP_2175	93177	92392		beta-lactamase
FNP_0581	941979	943874		beta-lactamase superfamily zinc-dependent hydrolase
FNP_0627	993909	992767		probable beta-lactamase superfamily zinc-dependent hydrolase
FNP_0629	995488	994865		beta-lactamase superfamily zinc-dependent hydrolase
*Outer membrane proteins*				
FNP_2182	98598	97345		probable lipoprotein
FNP_2196	112459	110366		outer membrane protein
FNP_2270	205012	203039		possible TonB-dependent outer membrane receptor
FNP_2283*	226990	219536		AT family autotransporter
FNP_2284	227596	227006		outer membrane protein
FNP_2361*	315873	308506		AT family autotransporter
FNP_2362	316452	315883		OmpA family outer membrane protein
FNP_0032	413132	404055		fusobacterial outer membrane protein
FNP_0217	583789	591255		fusobacterial outer membrane protein
FNP_0314*	693051	692284	*vacJ*	VacJ family lipoprotein
FNP_0378	751249	751797		outer membrane protein OmpF
FNP_0436	804352	805092		outer membrane protein
FNP_0509	874940	873669		TolC family outer membrane protein
FNP_0517	882518	883156		probable outer membrane protein
FNP_0668	1046781	1047941		OmpA family outer membrane protein
FNP_0820	1189564	1191015		possible outer membrane protein P1
FNP_0972*	1330393	1329281	*fomA*	porin FomA
FNP_1046*	1391420	1402057		AT family autotransporter
FNP_1248	1601352	1601074		OmpW
FNP_1784	2135004	2136413		outer membrane protein TolC
FNP_1877	2216275	2217012		possible outer membrane protein
FNP_1891	2231145	2236019		probable outer membrane protein
FNP_1996	2339205	2338783		probable lipoprotein
*Type IV secretion proteins*				
FNP_2389	342089	340542	*pulD*	general secretion pathway protein D
FNP_2396	346381	345881		A24 family prepilin peptidase
FNP_2397	346854	346266	*pulG*	general secretion protein G
FNP_2398	348121	347081	*pulG*	general secretion protein F
FNP_2399	349362	348118	*pulE*	general secretion protein E
FNP_1034	1383491	1382541	*pilT*	Tfp pilus assembly protein PilT
FNP_1868	2206819	2207523	*trbF*	probable conjugal transfer protein TrbF/VirB8
FNP_1869	2207535	2208371	*trbG*	probable conjugal transfer protein TrbG/VirB9
FNP_1870	2208381	2209583	*trbI*	probable conjugal transfer protein TrbI/VirB10
FNP_1871	2209586	2211616	*traG*	probable conjugal transfer protein TraG/VirD4
FNP_1873	2212045	2213010	*trbB*	probable conjugal transfer protein TrbB/VirB11
FNP_1875	2213293	2216031	*trbE*	probable conjugal transfer protein TrbE/VirB4
*Type V secretion proteins*				
FNP_2152	69523	69237		frameshift of AT family transporter
FNP_2283*	226990	219536		AT family autotransporter
FNP_2361*	315873	308506		AT family autotransporter
FNP_0035	417960	415012		possible autotransporter
FNP_1046*	1402057	1391420		AT family autotransporter
FNP_1391	1750650	1752584		possible autotransporter adhesin
FNP_1637	1986896	1998694		AT family autotransporter
FNP_2077	2423459	2420256		AT family autotransporter
FNP_0155*	520499	522103	*tpsB2*	probable TPS family two-partner secretion family protein TpsB
FNP_0156*	522114	530369	*tpsA2*	probable TPS family two-partner secretion family exoprotein TpsA
FNP_1246*	1599267	1589866	*tpsA1*	probable TPS family two-partner secretion exoprotein TpsA
FNP_1247*	1601074	1599281	*tpsB1*	pseudogene of TPS family two-partner secretion protein TpsB
*Proteases*				
FNP_0461	825985	827004		M50A family metalloprotease
FNP_0897	1266394	1267038		O-sialoglycoprotein endopeptidase
FNP_1813	2167021	2168046		O-sialoglycoprotein endopeptidase
*Lipopolysaccharide biosynthesis*				
FNP_2334	282271	280451		O-antigen acetylase
FNP_0533	898226	899365		possible ADP-heptose:LPS heptosyltransferase
FNP_0534	899377	900162	*licD*	lipopolysaccharide cholinephosphotransferase
FNP_0537	902005	903150	*waaG*	glycosyltransferase
FNP_0538	903150	903902		possible polysaccharide deacetylase
FNP_0539	903907	904767		possible glycosyltransferase
FNP_0540	904782	905864		possible glycosyltransferase
FNP_0541	905879	906610		probable glycosyltransferase
FNP_0544	908004	909200	*waaL*	possible O-antigen ligase
FNP_0830	1201261	1200185	*waaF1*	heptosyltransferase II (inner core)
FNP_1103	1443645	1442839		possible glycosyltransferase
FNP_1104	1444893	1443745		UDP-N-acetylglucosamine 2-epimerase
FNP_1105	1446154	1444898	*neuA*	CMP-N-acetylneuraminate cytidylyltransferase
FNP_1106	1447200	1446157	*neuB*	possible N-acetyl neuramic acid synthetase
FNP_1107	1447822	1447205		N-acetylneuraminate synthase
FNP_1108	1449226	1447934		oligosaccharidyl-lipid/polysaccharide flippase
FNP_1109	1450280	1449300		possible lipooligosaccharide sialyltransferase
FNP_1205	1556498	1557478	*waaE*	possible ADP-heptose synthase
FNP_1807	2161658	2162707	*waaF2*	LPS heptosyltransferase II
FNP_1808	2162704	2163732	*waaF3*	LPS heptosyltransferase II
FNP_1809	2163725	2164327	*waaY*	possible lipopolysaccharide core biosynthesis protein WaaY
FNP_1810	2164329	2165336	*waaQ*	lipopolysaccharide core biosynthesis glycosyl transferase WaaQ
FNP_1907	2251044	2251837	*lpxC*	UDP-3-O-acyl-N-acetylglucosamine deacetylase
FNP_1909	2252299	2253072	*lpxA*	acyl-[acyl-carrier-protein]–UDP-N-acetylglucosamine O-acyltransferase
FNP_1911	2253885	2254955	*lpxB*	lipid-A-disaccharide synthase

* Gene appears in more than one category within the table.

The acquisition of iron from the host environment is an important function of most bacterial pathogens [78]. We have identified 26 predicted proteins involved in iron uptake in FNP. Three proteins, HmuV (FNP_2267), HmuU (FNP_2266), and HmuT (FNP_2269), form a heme ABC superfamily ATP binding cassette transporter while two additional proteins (FNP_2270 and FNP_1765) are probable TonB-dependent heme receptors. There are also two additional iron ATP binding cassette transporters (FNP_428–430 and FNP_1451–1454) and a cobalamin/iron ATP binding cassette transporter (FNP_0398–0341). An Nramp family iron transporter (FNP_1660), and an OfeT family oxidase-dependent iron transporter (FNP_0531) were also annotated. Three hemolysin genes were identified (FNP_0006, FNP_0159, and FNP_0999); two of these (FNP_0159 and FNP_0999) have associated TPS family two-partner secretion proteins (FNP_0155/0156 and FNP_1246/1247, respectively). This is similar to what is seen in FNV, but different than FNN, which has three such pairs [21]. Several of the iron transporters (FNP_0339, FNP_0426, FNP_0428, FNP_0531, and FNP_0769) are present in FNN but are missing in FNV; thus FNV may have a diminished requirement for iron or may occupy a different niche. As mentioned previously, a homolog of the ferric uptake regulator, Fur (FNP_2353), was identified in the FNP genome, though it is not present in FNV.

Sixteen possible drug transporters were annotated. These included 7 MOP/MATE family multidrug efflux pumps (FNP_0174, FNP_0640, FNP_0890, FNP_1162, FNP_1207, FNP_1299, and FNP_1596), 2 DMT superfamily drug/metabolite transporters (FNP_0388 and FNP_0622) and 2 RND family antiporters (FNP_0507 and FNP_0508). Our annotation did not permit us to predict the substrates of these transporters but it is likely that many of them are antibiotic transporters. With respect to antibiotic resistance, we also annotated 4 genes predicted to encode beta-lactamases. One of these (FNP_0627) is unique to FNP.

We identified all but one (FN0387) of the 14 outer membrane protein genes described by Kapatral, et. al [20] (two, FNP_1046 and FNP_2283, have been re-annotated as AT family autotransporters) and we discovered a gene encoding OmpW (FNP_1248), which is not found in FNN or FNV. Four potential adhesion proteins were identified including a fibronectin-binding protein homolog (FNP_1337), a possible autotransporter adhesion (FNP_1391), and two proteins (FNP_1880 and FNP_1888) containing von Willebrand (vWF) type A domains. In addition to the Type IV secretion system discussed previously, FNP also carries genes that belong to the Type V secretion system. These include ten autotransporter genes (8 class 1, Type Va; 2 class 2, Type Vc) and the Tps secretion genes *tpsA* and *tpsB* (Type Vb) (Table 3). These are a subset of the genes found in FNN and FNV [62].

Twenty-five ORFs predicted to be involved in the biosynthesis of LPS were identified. This is of interest because *F. nucleatum* has been shown to have endotoxin activity [23], [79]. Unlike FNN, however, FNP does not possess the *lic* operon, which is predicted to attach choline residues to the LPS [20]; only the *licC* and *licD* genes, encoding phosphocholine cytidylyltransferase and a phosphotransferase, respectively, are present. In contrast, FNP does contain genes (FNP_1105–1107) that encode N-acylneuraminate cytidylyltransferase, N-acetyl neuraminate synthase, N-acetylneuraminate synthase and a possible lipooligosaccharide sialyltransferase (FNP_1109) that might incorporate sialic acid into LPS, like FNV (note, however, that FNP_1109 is not found in FNN or FNV). This may facilitate evasion from the host immune response.

### Signal Transduction

Six potential two-component signal transduction systems were revealed in FNP and three are of particular interest. An OmpR-related response regulator maps immediately upstream of a possible sensor histidine kinase gene. While the response regulator (FNP_2108) has homologs in both previously sequenced *F. nucleatum* genomes [20], [21] (FN1261, FNV1053), the sensor protein (FNP_2107) is not present in either of these two genomes. Also unique to this region is an ORF (FNP_2109) immediately downstream of the response regulator that is not found in the other sequenced fusobacterial genomes. This protein appears to contain both sensor histidine kinase and response regulator domains and may represent a fusion of two two-component domains. Another system is a possible ethanolamine two-component regulatory system (FNP_0128 and FNP_0129). This system is present in the FNN genome but is not found in the FNV genome, though it may be located in one of the unfinished regions of that genome. Both FNN and FNV have genes encoding the YesM/YesN two-component regulatory system. In FNP the response regulator, YesN (FNP_0212) has been interrupted by the insertion of an IStron, The IStron at this locus is predicted to be inactive so *yesN* should be non-functional. In addition to the six possible two-component systems identified in FNP, there is unmatched response regulator and one unmatched sensor histidine kinase.

### Communication with other bacteria

*F. nucleatum* plays an important role in the formation of oral biofilms, or plaque. *F. nucleatum* is believed to act as a bridging organism between the Gram-positive early colonizers and the Gram-negative late colonizers [23], [36], [70]. It has been proposed [80] that some bacteria use a compound known as AI-2 (autoinducer-2) for intra-species communication. AI-2 signaling is also involved with biofilm formation [81]. We have identified the protein responsible for AI-2 synthesis, LuxS (FNP_1558). LuxS activity has been previously reported in ATCC 10953 and in three additional *F. nucleatum* strains [82]. There is no homolog of *luxS* in either of the two previously annotated *F. nucleatum* genomes.

### Oxidative stress

Oral anaerobes must cope with oxidative stress to survive and contribute to pathogenesis. Established cultures of FNP are aerotolerent, indicating the presence of mechanisms to detoxify oxygen or oxygen radicals [83]. The ability of *F. nucleatum* to manage oxidative stress and to maintain reduced conditions is thought to facilitate the survival of other anaerobic pathogens. This facet of *F. nucleatum* ecology may explain why less aerotolerant organisms such as *Porphyromonas gingivalis* are increased in number in the presence of *F. nucleatum* [36]. Aeroprotection of bystander organisms may also explain the synergistic increase in virulence of *F. nucleatum* when combined with *Porphyromonas gingivalis*, as compared to either species alone [84], [85]. Physiological studies demonstrate that in response to oxidative stress, FNP maintains a reduced environment [5], with increases in NADH oxidase and superoxide dismutase activities [83]. Our analysis of the FNP genome revealed an NADH oxidase (FNP_1794), most closely related to treponemal and streptococcal homologs. An ORF encoding a superoxide dismutase was not identified, though, a rubrerythrin protein (FNP_1721), which confers superoxide dismutase-like activity [86] and has homology to a *C. perfringens* ruberythrin, is present in FNP. Other FNP proteins of potential importance in oxidative stress include a glutathione peroxidase (FNP_2310) [87], [88], a thioredoxin (FNP_0146) [89], a glutaredoxin (FNP_1273) [90], and an alkyl hydroperoxide (FNP_2288) [89], [91]. Orthologs of these genes are present in both the FNN and FNV genomes.

### Conclusion

Analysis of the genome sequence of *F. nucleatum* subsp. *polymorphum* revealed that this microorganism has obtained numerous genes from the *Firmicute* phylum of bacteria. In particular, many of the regions of FNP that were unique to the *Fusobacteria* encoded proteins with top BLAST hits to the *Clostridia*. This is perhaps not unexpected as *Firmicutes* were the largest phylotype isolated in a gingival bacterial diversity study, and the *Clostridia* represented the largest class within the *Firmicutes* that were isolated [1]. It appears that FNP was the recipient of DNA from bacteria that are common to the subgingival niche in HGT events. A contrasting interpretation, presented by Mira, *et al*. [92], is that *Fusobacterium* represents a genus with a *Clostridial* metabolic apparatus that has obtained a gram-negative envelope from the Proteobacteria. If this is the case, then it appears that FNN and FNV have lost a complement of genes that are present in FNP. As was revealed in the comparison of FNN to FNV, the fusobacterial genomes are mosaic in structure.

## Materials and Methods

### Bacterial strain, growth conditions, and DNA isolation

*F. nucleatum* subsp. *polymorphum* ATCC 10953 is a human strain originally isolated from the inflamed gingiva of an adult male [93]. The strain was acquired as a lyophil from the American Type Culture Collection and recovered on Columbia agar with 5% sheep blood under anaerobic growth conditions. Genomic DNA was prepared from an anaerobically cultivated Columbia broth culture, as previously described [71].

### Library construction and sequencing

Genomic DNA of *F. nucleatum* ATCC 10953 was sheared to 2–6 kb in size using a nebulizer (CIS-US, Inc., Bedford, Mass.), purified from an agarose gel, cloned into a pUC18 derivative sequencing vector, and sequenced as previous described [94].

### Sequence assembly

PHRED [95], [96] was used to determine the sequence and quality of each base of sequencing reads. Atlas genome assembly tools [97] were used to process reads, to remove repetitive reads, and to bin overlapping reads before assembling the contigs with the program PHRAP [95]. An initial assembly of 54,948 reads gave 146 contigs. Determination of linkages among contigs and gap closing was carried out as previously described [94] except that repetitive reads were put back into the contigs by separated bin assemblies. The rRNAs were not completely sequenced and there are 14 short gaps in the sequence.

The coordinate system for the genome was selected to be similar to other sequenced fusobacteria. In this system, the origin of replication is tentatively localized to the region upstream of an ORF with low identity to DnaA. *recF* and *gyrB* are found downstream of this putative *dnaA* gene. *dnaE* and *repB*, which are often clustered with *dnaA* around the origin of replication, are found elsewhere in the FNP genome, as they are in the other two *Fusobacterium* genomes

### Gene identification and annotation

Gene prediction and manual annotation were performed as previously described [94]. For GeneMark [98] gene predictions, *Borrelia burgdorferi* was used as model. Predicted proteins with unknown functions were classified into three categories: “hypothetical protein” for proteins that do not have matches in the GenBank NR database, “conserved hypothetical protein” for proteins that have matches to proteins of unknown functions from organisms outside the genus *Fusobacterium*, and “fusobacterial conserved hypothetical protein” for proteins that have matches only to other fusobacterial proteins of unknown functions.

Transfer RNAs were identified using tRNAscan-SE [99]. Non-coding RNA (ncRNA) elements where identified by BLASTing the known ncRNA sequences of FNN, available at the Rfam database (http://www.sanger.ac.uk/Software/Rfam/), against the FNP genome [100]. The FNP regions containing potential ncRNAs were then analyzed by the Rfam database to identify the exact coordinates of each RNA element.

### Genome analysis and identification of possible HGT regions

We performed automatic and manual comparison of best hits to the GenBank NR database and BLAST results to the FNN and FNV genomes to find possible genomic islands and recent horizontally transferred genes in the FNP genome. Genes and regions of possible HGT were examined by GC content, cumulative GC profile, and codon usage bias analysis [101] but this did not add significantly to the conclusions. ClustalW was also used to align DNA sequences and to identify repeat sequences in the genome [102].

### Analysis of intergenic regions

After completing annotation of all ORFs called by either GeneMark or Glimmer we decided to confirm that the intergenic regions (IGR) contained no ORFs missed by the ORF calling software. Thus, the entire nucleotide sequence of each IGR was compared to Genbank using BLASTX and those results that had an e value of less than 1×10^−5^ were marked for further analysis by annotators. The annotators defined the exact coordinates of each hit and then followed the same process used for the called ORFs to assign the annotation for each region, resulting in the addition of 50 annotation entries. While this process did result in the identification of a few missed ORFs (5–10), the vast majority of IGR annotations were pseudogenes. Of note concerning this analysis is that it allowed us to extend proteins called as small hypothetical proteins into full-length pseudogenes.

### Database submission

The FNP ATCC 10953 genome sequence was deposited in GenBank and assigned accession number CM000440 and the plasmid was assigned accession number CP000710.

## Supporting Information

Table S1 FNP ORFS not in FNN or FNV; FNP ORFS not in FNN; and FNP ORFS not found in FNV.
(1.17 MB DOC)

Table S2 Clusters of ORFs unique to FNP and not in FNN or FNV.
(0.74 MB DOC)
