# Supplementary material for: Genome Sequence of Fusobacterium nucleatum Subspecies Polymorphum — a Genetically Tractable Fusobacterium
Source: PLoS One. 2007 Aug 1;2(8):e659. doi: 10.1371/journal.pone.0000659 (PMC1924603; doi:10.1371/journal.pone.0000659)
Supplement: Table S1 — FNP ORFS not in FNN or FNV; FNP ORFS not in FNN; and FNP ORFS not found in FNV. (1.17 MB DOC) [file pone.0000659.s001.doc]

**Table S1a. FNP ORFS not in FNN or FNV**

| **Start** | **Stop** | **Locus Tag** | **Definition** |
| --- | --- | --- | --- |
| 1244 | 588 | FNP_2086 | hypothetical protein |
| 6458 | 7174 | FNP_2091 | hypothetical protein |
| 7231 | 7464 | FNP_2092 | hypothetical protein |
| 23482 | 22685 | FNP_2107 | possible sensor histidine kinase |
| 24166 | 23495 | FNP_2108 | probable OmpR family response regulator |
| 26044 | 24173 | FNP_2109 | bifunctional response regulator/sensor histidine kinase |
| 26171 | 27280 | FNP_2110 | alcohol dehydrogenase |
| 27349 | 27465 | FNP_2111 | hypothetical protein |
| 27598 | 28407 | FNP_2112 | propanediol utilization protein |
| 28428 | 31166 | FNP_2113 | pseudogene of glycerol dehydratase, large subunit |
| 31183 | 31863 | FNP_2115 | propanediol dehydratase, medium subunit |
| 31877 | 32386 | FNP_2116 | propanediol dehydratase, small subunit |
| 32409 | 34223 | FNP_2117 | propanediol dehydratase, large subunit |
| 34223 | 34600 | FNP_2118 | conserved hypothetical protein |
| 35374 | 35994 | FNP_2121 | propanediol utilization protein |
| 36008 | 36718 | FNP_2122 | conserved hypothetical protein |
| 37013 | 37954 | FNP_2124 | conserved hypothetical protein |
| 37970 | 39367 | FNP_2125 | probable aldehyde dehydrogenase |
| 75849 | 74701 | FNP_2158 | probable alcohol dehydrogenase |
| 77748 | 77665 | FNP_2160 | hypothetical protein |
| 96367 | 96254 | FNP_2179 | hypothetical protein |
| 124792 | 124139 | FNP_2205 | conserved hypothetical protein |
| 125850 | 124792 | FNP_2206 | possible ATP-binding protein |
| 128759 | 125970 | FNP_2207 | conserved hypothetical protein |
| 131810 | 128760 | FNP_2208 | conserved hypothetical protein |
| 135156 | 131824 | FNP_2209 | conserved hypothetical protein |
| 138609 | 135166 | FNP_2210 | conserved hypothetical protein |
| 139951 | 138641 | FNP_2211 | hypothetical protein |
| 140538 | 140002 | FNP_2212 | hypothetical protein |
| 141164 | 140556 | FNP_2213 | hypothetical protein |
| 141827 | 141180 | FNP_2214 | hypothetical protein |
| 145221 | 141850 | FNP_2215 | conserved hypothetical protein |
| 146059 | 145232 | FNP_2216 | possible transcriptional regulator |
| 146485 | 146273 | FNP_2217 | hypothetical protein |
| 157802 | 157563 | FNP_2227 | probable transposase |
| 175562 | 174909 | FNP_2242 | hypothetical protein |
| 176281 | 175574 | FNP_2243 | hypothetical protein |
| 177511 | 176312 | FNP_2244 | probable hydrolase |
| 182334 | 182221 | FNP_2247 | hypothetical protein |
| 188201 | 187440 | FNP_2254 | hypothetical protein |
| 188708 | 188502 | FNP_2256 | hypothetical protein |
| 198622 | 198041 | FNP_2264 | hypothetical protein |
| 255474 | 254509 | FNP_2311 | dehydrogenase |
| 262220 | 261375 | FNP_2316 | conserved hypothetical protein |
| 264111 | 263182 | FNP_2318 | conserved hypothetical protein |
| 265045 | 264089 | FNP_2319 | conserved hypothetical protein |
| 279801 | 279376 | FNP_2332 | hypothetical protein |
| 279590 | 280153 | FNP_2333 | bifunctional diaminohydroxyphosphoribosylaminopyrimidine deaminase/ 5-amino-6-(5-phosphoribosylamino)uracil reductase |
| 282448 | 282350 | FNP_2335 | hypothetical protein |
| 287556 | 287410 | FNP_2341 | hypothetical protein |
| 299807 | 299214 | FNP_2352 | hypothetical protein |
| 301107 | 302276 | FNP_2355 | possible transposase |
| 302825 | 302649 | FNP_2356 | hypothetical protein |
| 303742 | 302822 | FNP_2357 | possible periplasmic serine protease |
| 325734 | 325630 | FNP_2374 | hypothetical protein |
| 332113 | 332021 | FNP_2381 | hypothetical protein |
| 343130 | 343047 | FNP_2391 | hypothetical protein |
| 350919 | 350482 | FNP_2402 | possible histone acetyltransferase |
| 351489 | 350944 | FNP_2403 | probable NADPH oxidoreductase |
| 351748 | 352176 | FNP_2404 | Tn*10* family transposase |
| 352243 | 352395 | FNP_2405 | hypothetical protein |
| 356599 | 356829 | FNP_2410 | hypothetical protein |
| 356819 | 357103 | FNP_2411 | possible plasmid addiction system protein |
| 359088 | 359921 | FNP_2413 | hypothetical protein |
| 363090 | 363206 | FNP_2417 | hypothetical protein |
| 363181 | 363792 | FNP_2418 | possible transcriptional regulator |
| 369888 | 371375 | FNP_2423 | AGCS family alanine:sodium (Na+) symporter carrier |
| 385061 | 385153 | FNP_0002 | hypothetical protein |
| 385365 | 385255 | FNP_0003 | hypothetical protein |
| 385497 | 385631 | FNP_0004 | hypothetical protein |
| 391253 | 391444 | FNP_0011 | hypothetical protein |
| 395014 | 395211 | FNP_0016 | possible DNA-damage-inducible protein J |
| 395411 | 395647 | FNP_0018 | possible TnpX family recombinase |
| 395735 | 395947 | FNP_0019 | conserved hypothetical protein |
| 396807 | 398111 | FNP_0021 | conserved hypothetical protein |
| 398130 | 398282 | FNP_0022 | hypothetical protein |
| 398338 | 398466 | FNP_0023 | hypothetical protein |
| 398703 | 398894 | FNP_0024 | conserved hypothetical protein |
| 400058 | 399939 | FNP_0026 | hypothetical protein |
| 401349 | 400846 | FNP_0028 | conserved hypothetical protein |
| 417960 | 415012 | FNP_0035 | possible autotransporter |
| 424671 | 425297 | FNP_0045 | hypothetical protein |
| 425594 | 425968 | FNP_0046 | hypothetical protein |
| 426382 | 426723 | FNP_0047 | hypothetical protein |
| 426749 | 427312 | FNP_0048 | hypothetical protein |
| 427349 | 428290 | FNP_0049 | hypothetical protein |
| 428334 | 428744 | FNP_0050 | hypothetical protein |
| 428961 | 429293 | FNP_0051 | hypothetical protein |
| 429942 | 430340 | FNP_0053 | hypothetical protein |
| 430412 | 430978 | FNP_0054 | hypothetical protein |
| 432494 | 432595 | FNP_0058 | hypothetical protein |
| 432804 | 434465 | FNP_0059 | dihydroxy-acid dehydratase |
| 435961 | 437682 | FNP_0061 | acetolactate synthase large subunit |
| 437675 | 438163 | FNP_0062 | acetolactate synthase small subunit |
| 438406 | 439914 | FNP_0063 | 2-isopropylmalate synthase |
| 439925 | 441316 | FNP_0064 | 3-isopropylmalate dehydratase large subunit |
| 441316 | 441891 | FNP_0065 | 3-isopropylmalate dehydratase small subunit |
| 442032 | 442148 | FNP_0066 | hypothetical protein |
| 442203 | 443261 | FNP_0067 | 3-isopropylmalate dehydrogenase |
| 443492 | 444277 | FNP_0068 | ABC superfamily ATP binding cassette transporter, membrane/binding protein |
| 444488 | 445495 | FNP_0069 | ketol-acid reductoisomerase |
| 452842 | 452600 | FNP_0073 | conserved hypothetical protein |
| 454422 | 453874 | FNP_0075 | hypothetical protein |
| 459152 | 460696 | FNP_0082 | AbgT family p-aminobenzoyl-glutamate transporter |
| 461581 | 460769 | FNP_0083 | conserved hypothetical protein |
| 462101 | 461571 | FNP_0084 | hypothetical protein |
| 482786 | 482953 | FNP_0106 | hypothetical protein |
| 484507 | 484902 | FNP_0109 | hypothetical protein |
| 484899 | 485051 | FNP_0110 | hypothetical protein |
| 485061 | 485765 | FNP_0111 | hypothetical protein |
| 485810 | 486151 | FNP_0112 | hypothetical protein |
| 486167 | 486670 | FNP_0113 | hypothetical protein |
| 487905 | 487792 | FNP_0116 | hypothetical protein |
| 504477 | 504581 | FNP_0130 | hypothetical protein |
| 518631 | 518813 | FNP_0147 | conserved hypothetical protein |
| 518870 | 518938 | FNP_0148 | hypothetical protein |
| 519191 | 518985 | FNP_0149 | conserved hypothetical protein |
| 519235 | 519333 | FNP_0150 | conserved hypothetical protein |
| 519485 | 519330 | FNP_0151 | hypothetical protein |
| 520248 | 520349 | FNP_0154 | hypothetical protein |
| 545409 | 546161 | FNP_0173 | conserved hypothetical protein |
| 547769 | 548635 | FNP_0175 | 5,10-methylenetetrahydrofolate reductase (FADH) |
| 553012 | 553125 | FNP_0178 | hypothetical protein |
| 553129 | 553626 | FNP_0179 | probable acetyltransferase |
| 553700 | 554470 | FNP_0180 | conserved hypothetical protein |
| 555338 | 555904 | FNP_0183 | conserved hypothetical protein |
| 556006 | 556134 | FNP_0184 | hypothetical protein |
| 556159 | 556356 | FNP_0185 | conserved hypothetical protein |
| 562524 | 562432 | FNP_0191 | hypothetical protein |
| 564271 | 564396 | FNP_0195 | hypothetical protein |
| 565168 | 564488 | FNP_0196 | probable transcriptional regulator |
| 566230 | 565265 | FNP_0197 | possible dehydrogenase |
| 567516 | 566245 | FNP_0198 | probable C4-dicarboxylate TRAP-T family tripartite ATP-independent periplasmic transporter, membrane protein, large subunit |
| 568004 | 567528 | FNP_0199 | probable C4-dicarboxylater TRAP-T family tripartite ATP-independent periplasmic transporter, membrane protein, small subunit |
| 569035 | 568025 | FNP_0200 | probable TRAP-T family tripartite ATP-independent periplasmic C4-dicarboxylate transporter, binding protein |
| 569457 | 569050 | FNP_0201 | conserved hypothetical protein |
| 570665 | 569460 | FNP_0202 | conserved hypothetical protein |
| 574605 | 574090 | FNP_0206 | conserved hypothetical protein |
| 575272 | 574619 | FNP_0207 | conserved hypothetical protein |
| 579972 | 579673 | FNP_0214 | hypothetical protein |
| 580132 | 581235 | FNP_0215 | transposase |
| 604748 | 605827 | FNP_0230 | probable histidine kinase |
| 606514 | 605912 | FNP_0231 | conserved hypothetical protein |
| 606787 | 607494 | FNP_0232 | possible DeoR family transcriptional regulator |
| 607607 | 609154 | FNP_0233 | hypothetical protein |
| 609192 | 611585 | FNP_0234 | possible ATPase |
| 611620 | 612750 | FNP_0235 | conserved hypothetical protein |
| 613050 | 613271 | FNP_0236 | hypothetical protein |
| 614968 | 614342 | FNP_0240 | possible transposase |
| 627090 | 627254 | FNP_0253 | hypothetical protein |
| 636577 | 636828 | FNP_0263 | hypothetical protein |
| 636977 | 637123 | FNP_0264 | hypothetical protein |
| 637175 | 637675 | FNP_0265 | conserved hypothetical protein |
| 638040 | 638690 | FNP_0266 | hypothetical protein |
| 638758 | 639483 | FNP_0267 | hypothetical protein |
| 639501 | 640205 | FNP_0268 | hypothetical protein |
| 653684 | 660322 | FNP_0284 | probable outer membrane protein |
| 663326 | 662040 | FNP_0286 | O-acetylhomoserine aminocarboxypropyltransferase |
| 681084 | 681779 | FNP_0302 | aspartate racemase |
| 681938 | 682660 | FNP_0303 | ABC superfamily ATP binding cassette transporter, membrane protein |
| 682641 | 683321 | FNP_0304 | ABC superfamily ATP binding cassette transporter, membrane protein |
| 684091 | 684969 | FNP_0306 | ABC superfamily ATP binding cassette transporter, binding protein |
| 685518 | 685033 | FNP_0307 | conserved hypothetical protein |
| 686518 | 685580 | FNP_0308 | probable transcriptional regulator |
| 687055 | 688203 | FNP_0310 | cystathionine gamma-synthase |
| 702587 | 702519 | FNP_0324 | hypothetical protein |
| 703137 | 702607 | FNP_0325 | hypothetical protein |
| 703707 | 703138 | FNP_0326 | conserved hypothetical protein |
| 711185 | 712822 | FNP_0333 | possible dipeptide/oligopeptide/nickel (Ni2+) ABC superfamily ATP binding cassette transporter, binding protein |
| 712851 | 713828 | FNP_0334 | possible dipeptide/oligopeptide/nickel (Ni2+) ABC superfamily ATP binding cassette transporter, membrane protein |
| 715542 | 716156 | FNP_0337 | dipeptide/oligopeptide/nickel (Ni2+) ABC superfamily ATP binding cassette transporter, ABC protein |
| 724125 | 724427 | FNP_0345 | hypothetical protein |
| 724448 | 724759 | FNP_0346 | hypothetical protein |
| 724831 | 725667 | FNP_0347 | conserved hypothetical protein |
| 731133 | 731041 | FNP_0353 | hypothetical protein |
| 731354 | 731710 | FNP_0354 | conserved hypothetical protein |
| 736584 | 736970 | FNP_0361 | possible transcriptional regulator |
| 736972 | 737979 | FNP_0362 | possible flavin-dependent oxidoreductase |
| 741703 | 741942 | FNP_0366 | hypothetical protein |
| 741944 | 742792 | FNP_0367 | anititoxin/toxin system zeta toxin |
| 751850 | 751794 | FNP_0379 | hypothetical protein |
| 768143 | 768844 | FNP_0397 | hypothetical protein |
| 769162 | 769899 | FNP_0398 | hypothetical protein |
| 770062 | 770787 | FNP_0399 | hypothetical protein |
| 770914 | 771693 | FNP_0400 | conserved hypothetical protein |
| 771727 | 772839 | FNP_0401 | hypothetical protein |
| 772849 | 773790 | FNP_0402 | conserved hypothetical protein |
| 773817 | 774539 | FNP_0403 | hypothetical protein |
| 774536 | 775498 | FNP_0404 | conserved hypothetical protein |
| 776194 | 776078 | FNP_0406 | hypothetical protein |
| 777458 | 776241 | FNP_0407 | transposase |
| 784438 | 784584 | FNP_0417 | hypothetical protein |
| 805107 | 805526 | FNP_0437 | hypothetical protein |
| 805595 | 806578 | FNP_0438 | conserved hypothetical protein |
| 814537 | 814638 | FNP_0446 | hypothetical protein |
| 814778 | 814960 | FNP_0447 | hypothetical protein |
| 860352 | 860465 | FNP_0496 | conserved hypothetical protein |
| 867423 | 866884 | FNP_0504 | possible phosphodiesterase |
| 894140 | 894343 | FNP_0529 | hypothetical protein |
| 910078 | 909221 | FNP_0545 | probable ADP-ribosyl-[dinitrogen reductase] hydrolase |
| 913057 | 911639 | FNP_0548 | beta-fructofuranosidase |
| 917610 | 917296 | FNP_0552 | hypothetical protein |
| 925018 | 924194 | FNP_0561 | possible amidohydrolase |
| 925123 | 925890 | FNP_0562 | hypothetical protein |
| 925974 | 926066 | FNP_0563 | hypothetical protein |
| 932205 | 933356 | FNP_0570 | probable transposase |
| 933475 | 933678 | FNP_0571 | conserved hypothetical protein |
| 961145 | 962689 | FNP_0601 | nickel (Ni2+) ABC superfamily ATP binding cassette transporter, binding protein |
| 962784 | 963545 | FNP_0602 | pseudogene of nickel (Ni2+) ABC superfamily ATP binding cassette transporter, membrane protein |
| 963542 | 964366 | FNP_0603 | nickel (Ni2+) ABC superfamily ATP binding cassette transporter, membrane protein |
| 964366 | 965172 | FNP_0604 | oligopeptide ABC superfamily ATP binding cassette transporter, ABC protein |
| 965162 | 965782 | FNP_0605 | oligopeptide ABC superfamily ATP binding cassette transporter, ABC protein |
| 965787 | 965927 | FNP_0606 | hypothetical protein |
| 993909 | 992767 | FNP_0627 | probable beta-lactamase superfamily zinc (Zn2+)-dependent hydrolase |
| 1019386 | 1019874 | FNP_0645 | conserved hypothetical protein |
| 1022627 | 1022532 | FNP_0648 | hypothetical protein |
| 1042107 | 1041712 | FNP_0664 | possible acetyltransferase |
| 1056861 | 1056490 | FNP_0677 | hypothetical protein |
| 1057780 | 1057676 | FNP_0679 | hypothetical protein |
| 1072096 | 1072305 | FNP_0694 | hypothetical protein |
| 1073058 | 1072660 | FNP_0696 | hypothetical protein |
| 1073860 | 1073177 | FNP_0697 | hypothetical protein |
| 1074467 | 1073847 | FNP_0698 | hypothetical protein |
| 1075682 | 1074684 | FNP_0699 | hypothetical protein |
| 1075965 | 1075651 | FNP_0700 | conserved hypothetical protein |
| 1078947 | 1081319 | FNP_0703 | possible helicase |
| 1089648 | 1089106 | FNP_0711 | conserved hypothetical protein |
| 1096571 | 1096476 | FNP_0722 | hypothetical protein |
| 1124734 | 1123592 | FNP_0754 | conserved hypothetical protein |
| 1128220 | 1126529 | FNP_0757 | conserved hypothetical protein |
| 1128990 | 1128250 | FNP_0758 | hypothetical protein |
| 1129842 | 1129039 | FNP_0759 | conserved hypothetical protein |
| 1129967 | 1130305 | FNP_0760 | probable transcriptional regulator |
| 1131073 | 1130309 | FNP_0761 | hypothetical protein |
| 1132640 | 1132113 | FNP_0763 | hypothetical protein |
| 1135725 | 1134934 | FNP_0767 | probable hydrolase |
| 1136178 | 1135747 | FNP_0768 | probable MarR family transcriptional regulator |
| 1137526 | 1139106 | FNP_0770 | possible ferredoxin-nitrite reductase |
| 1139107 | 1140915 | FNP_0771 | conserved hypothetical protein |
| 1140929 | 1141681 | FNP_0772 | possible nitrate/sulfonate/bicarbonate ABC superfamily ATP binding cassette transporter, membrane protein |
| 1141694 | 1142455 | FNP_0773 | probable nitrate/sulfonate/bicarbonate ABC superfamily ATP binding cassette transporter, ABC protein |
| 1142477 | 1143514 | FNP_0774 | possible nitrate/sulfonate/bicarbonate ABC superfamily ATP binding cassette transporter, binding protein |
| 1160759 | 1160004 | FNP_0792 | conserved hypothetical protein |
| 1170096 | 1170902 | FNP_0802 | hypothetical protein |
| 1171613 | 1172017 | FNP_0804 | conserved hypothetical protein |
| 1172001 | 1172384 | FNP_0805 | conserved hypothetical protein |
| 1172385 | 1172462 | FNP_0806 | hypothetical protein |
| 1172989 | 1174293 | FNP_0808 | iron-sulfur (Fe-S) oxidoreductase |
| 1174290 | 1177193 | FNP_0809 | pseudogene of iron-sulfur (Fe-S) oxidoreductase |
| 1177200 | 1179365 | FNP_0810 | conserved hypothetical protein |
| 1180378 | 1179515 | FNP_0811 | possible lauroyl acyltransferase |
| 1180625 | 1181350 | FNP_0812 | possible zinc (Zn2+)-dependent hydrolase/metallo-beta-lactamase |
| 1181343 | 1182248 | FNP_0813 | hypothetical protein |
| 1182264 | 1182962 | FNP_0814 | conserved hypothetical protein |
| 1182977 | 1184524 | FNP_0815 | conserved hypothetical protein |
| 1184521 | 1185849 | FNP_0816 | possible iron-sulfur (Fe-S) oxidoreductase |
| 1187331 | 1185892 | FNP_0817 | APC family amino acid-polyamine-organocation transporter, glutamate:gamma-aminobutyrate antiporter |
| 1188730 | 1187351 | FNP_0818 | glutamate decarboxylase |
| 1191015 | 1189564 | FNP_0820 | possible outer membrane protein P1 |
| 1198601 | 1198717 | FNP_0828 | hypothetical protein |
| 1204258 | 1202195 | FNP_0832 | hypothetical protein |
| 1205493 | 1204264 | FNP_0833 | hypothetical protein |
| 1205793 | 1205722 | FNP_0834 | hypothetical protein |
| 1206173 | 1205802 | FNP_0835 | hypothetical protein |
| 1206420 | 1206145 | FNP_0836 | hypothetical protein |
| 1206466 | 1206356 | FNP_0837 | hypothetical protein |
| 1215463 | 1215906 | FNP_0845 | hypothetical protein |
| 1218905 | 1219936 | FNP_0848 | conserved hypothetical protein |
| 1230586 | 1231152 | FNP_0859 | conserved hypothetical protein |
| 1234657 | 1235895 | FNP_0864 | probable AAA+ superfamily ATPase |
| 1240140 | 1240526 | FNP_0870 | conserved hypothetical protein |
| 1242802 | 1243704 | FNP_0875 | hypothetical protein |
| 1267226 | 1269244 | FNP_0898 | conserved hypothetical protein |
| 1271722 | 1271621 | FNP_0902 | hypothetical protein |
| 1275174 | 1275064 | FNP_0906 | hypothetical protein |
| 1287746 | 1287021 | FNP_0922 | probable ABC superfamily ATP binding cassette transporter, ABC protein |
| 1288453 | 1287671 | FNP_0923 | probable nitrate/sulfonate/bicarbonate ABC superfamily ATP binding cassette transporter, membrane protein |
| 1289378 | 1288428 | FNP_0924 | probable nitrate/sulfonate/bicarbonate ABC superfamily ATP binding cassette transporter, binding protein |
| 1289899 | 1289705 | FNP_0925 | hypothetical protein |
| 1289990 | 1289778 | FNP_0926 | hypothetical protein |
| 1297585 | 1297358 | FNP_0935 | possible DNA-damage-inducible protein RelB |
| 1307800 | 1307874 | FNP_0947 | hypothetical protein |
| 1308488 | 1308973 | FNP_0949 | transposase-like protein B |
| 1309247 | 1308945 | FNP_0950 | hypothetical protein |
| 1309250 | 1309594 | FNP_0951 | transposase-like protein B |
| 1315198 | 1316475 | FNP_0959 | glycosyltransferase |
| 1332327 | 1332235 | FNP_0974 | hypothetical protein |
| 1342214 | 1342430 | FNP_0984 | hypothetical protein |
| 1342496 | 1342404 | FNP_0985 | hypothetical protein |
| 1345427 | 1344078 | FNP_0988 | possible transcriptional regulator |
| 1351423 | 1351334 | FNP_1001 | hypothetical protein |
| 1358853 | 1358990 | FNP_1009 | hypothetical protein |
| 1361219 | 1360233 | FNP_1011 | conserved hypothetical protein |
| 1362373 | 1361270 | FNP_1013 | transposase-like protein B |
| 1379183 | 1379449 | FNP_1029 | hypothetical protein |
| 1379614 | 1379712 | FNP_1030 | hypothetical protein |
| 1385911 | 1386036 | FNP_1037 | hypothetical protein |
| 1415453 | 1415953 | FNP_1074 | hypothetical protein |
| 1419222 | 1418413 | FNP_1077 | possible M48B family peptidase |
| 1425108 | 1426016 | FNP_1083 | hypothetical protein |
| 1426055 | 1426537 | FNP_1084 | hypothetical protein |
| 1428290 | 1428718 | FNP_1088 | possible hydrolase |
| 1428739 | 1429194 | FNP_1089 | hypothetical protein |
| 1432327 | 1430930 | FNP_1091 | hypothetical protein |
| 1433721 | 1432342 | FNP_1092 | possible GUP family alanine uptake protein |
| 1436046 | 1435852 | FNP_1096 | hypothetical protein |
| 1436149 | 1436033 | FNP_1097 | hypothetical protein |
| 1436693 | 1436271 | FNP_1098 | hypothetical protein |
| 1439611 | 1436696 | FNP_1099 | hypothetical protein |
| 1440187 | 1439630 | FNP_1100 | possible phosphatase |
| 1442105 | 1440174 | FNP_1101 | conserved hypothetical protein |
| 1442858 | 1442115 | FNP_1102 | possible phosphoribosyltransferase |
| 1443645 | 1442839 | FNP_1103 | probable glycosyltransferase |
| 1449226 | 1447934 | FNP_1108 | MOP superfamily multidrug/oligosaccharide-lipid/polysaccharide flippase |
| 1450280 | 1449300 | FNP_1109 | possible lipooligosaccharide sialyltransferase |
| 1451710 | 1450283 | FNP_1110 | hypothetical protein |
| 1453770 | 1452559 | FNP_1112 | conserved hypothetical protein |
| 1470823 | 1472529 | FNP_1129 | conserved hypothetical protein |
| 1472638 | 1473918 | FNP_1130 | hypothetical protein |
| 1473923 | 1475032 | FNP_1131 | possible AAA family ATPase |
| 1475748 | 1479764 | FNP_1132 | possible helicase |
| 1479761 | 1482217 | FNP_1133 | possible ATPase |
| 1482227 | 1483357 | FNP_1134 | hypothetical protein |
| 1483357 | 1485207 | FNP_1135 | possible S14 family endopeptidase ClpA |
| 1485216 | 1485782 | FNP_1136 | [formate-C-acetyltransferase]-activating enzyme |
| 1486017 | 1486100 | FNP_1137 | hypothetical protein |
| 1486167 | 1487249 | FNP_1138 | hypothetical protein |
| 1487246 | 1488007 | FNP_1139 | possible [formate-C-acetyltransferase]-activating enzyme |
| 1488019 | 1489089 | FNP_1140 | possible iron-sulfur (Fe-S) oxidoreductase |
| 1489067 | 1490296 | FNP_1141 | radical SAM superfamily protein |
| 1490281 | 1491795 | FNP_1142 | probable acetolactate synthase |
| 1491806 | 1492303 | FNP_1143 | adenylyl-sulfate kinase |
| 1492307 | 1493059 | FNP_1144 | methyltransferase |
| 1493064 | 1495379 | FNP_1145 | possible bifunctional phosphoenolpyruvate synthase/pyruvate phosphate dikinase |
| 1495373 | 1495954 | FNP_1146 | possible glutamine amidotransferase |
| 1495951 | 1496700 | FNP_1147 | probable sugar nucleotidyltransferase |
| 1496706 | 1497518 | FNP_1148 | probable PHP family metal-dependent phosphoesterase |
| 1498509 | 1498153 | FNP_1149 | possible transposase |
| 1498767 | 1499162 | FNP_1150 | conserved hypothetical protein |
| 1499174 | 1499764 | FNP_1151 | probable transcriptional regulator |
| 1507572 | 1508246 | FNP_1157 | hypothetical protein |
| 1534745 | 1530747 | FNP_1179 | conserved hypothetical protein |
| 1560365 | 1559382 | FNP_1208 | possible site-specific deoxyribonuclease |
| 1561840 | 1560362 | FNP_1209 | DNA (cytosine-5-)-methyltransferase |
| 1562899 | 1563015 | FNP_1212 | hypothetical protein |
| 1563476 | 1564720 | FNP_1213 | hypothetical protein |
| 1563476 | 1564720 | FNP_1214 | probable transposase |
| 1565794 | 1565919 | FNP_1216 | hypothetical protein |
| 1568683 | 1568594 | FNP_1220 | hypothetical protein |
| 1586066 | 1585647 | FNP_1238 | hypothetical protein |
| 1586656 | 1586096 | FNP_1239 | conserved hypothetical protein |
| 1586683 | 1586955 | FNP_1240 | hypothetical protein |
| 1587253 | 1586939 | FNP_1241 | hypothetical protein |
| 1587728 | 1587333 | FNP_1242 | hypothetical protein |
| 1588474 | 1587752 | FNP_1243 | conserved hypothetical protein |
| 1588957 | 1588622 | FNP_1244 | hypothetical protein |
| 1589869 | 1589240 | FNP_1245 | hypothetical protein |
| 1601352 | 1601987 | FNP_1248 | possible outer membrane protein OmpW |
| 1621353 | 1622114 | FNP_1269 | ABC superfamily ATP binding cassette transporter, binding protein |
| 1624846 | 1624283 | FNP_1272 | flavin oxidoreductase domain protein |
| 1631187 | 1630846 | FNP_1276 | hypothetical protein |
| 1633846 | 1633268 | FNP_1279 | DNA-3-methyladenine glycosylase I |
| 1637508 | 1636192 | FNP_1282 | possible transcriptional regulator |
| 1638932 | 1637577 | FNP_1283 | possible transcriptional regulator |
| 1640340 | 1638955 | FNP_1285 | possible transcriptional regulator |
| 1656513 | 1654798 | FNP_1301 | transcriptional regulator |
| 1656760 | 1657968 | FNP_1302 | threonine ammonia-lyase |
| 1657981 | 1659165 | FNP_1303 | acetylornithine deacetylase/succinyl-diaminopimelate |
| 1659224 | 1660696 | FNP_1304 | SSS family solute:sodium (Na+) symporter |
| 1660710 | 1662299 | FNP_1305 | N-acyl-D-aspartate/D-glutamate deacylase |
| 1668533 | 1670029 | FNP_1312 | type I site-specific deoxyribonuclease, methyltransferase subunit |
| 1670016 | 1671179 | FNP_1313 | type I site-specific deoxyribonuclease, specificity subunit |
| 1671176 | 1672312 | FNP_1314 | type I site-specific deoxyribonuclease, restriction subunit |
| 1672401 | 1672628 | FNP_1315 | hypothetical protein |
| 1672601 | 1672717 | FNP_1316 | hypothetical protein |
| 1672874 | 1672954 | FNP_1317 | hypothetical protein |
| 1673146 | 1674393 | FNP_1318 | possible transposase |
| 1674459 | 1674605 | FNP_1319 | hypothetical protein |
| 1674590 | 1674901 | FNP_1320 | conserved hypothetical protein |
| 1674945 | 1675934 | FNP_1321 | possible bacteriophage integrase/recombinase |
| 1675995 | 1679378 | FNP_1322 | type I site-specific deoxyribonuclease, restriction subunit |
| 1680938 | 1681714 | FNP_1324 | conserved hypothetical protein |
| 1683911 | 1683024 | FNP_1327 | ABC superfamily ATP binding cassette transporter, membrane protein |
| 1684962 | 1683943 | FNP_1328 | ABC superfamily ATP binding cassette transporter, binding protein |
| 1687418 | 1688524 | FNP_1331 | hypothetical protein |
| 1688499 | 1689182 | FNP_1332 | conserved hypothetical protein |
| 1737989 | 1737606 | FNP_1375 | hypothetical protein |
| 1738339 | 1738013 | FNP_1376 | hypothetical protein |
| 1740787 | 1739726 | FNP_1379 | conserved hypothetical protein |
| 1742967 | 1742401 | FNP_1382 | pseudogene of conserved hypothetical protein |
| 1743143 | 1742967 | FNP_1383 | hypothetical protein |
| 1747311 | 1746496 | FNP_1388 | conserved hypothetical protein |
| 1753891 | 1754118 | FNP_1394 | hypothetical protein |
| 1755276 | 1754185 | FNP_1395 | aspartate-semialdehyde dehydrogenase |
| 1756141 | 1755257 | FNP_1396 | homoserine kinase ThrB |
| 1757586 | 1756129 | FNP_1397 | threonine synthase |
| 1757682 | 1758812 | FNP_1398 | homoserine dehydrogenase |
| 1758812 | 1760128 | FNP_1399 | aspartate kinase |
| 1762504 | 1761959 | FNP_1402 | conserved hypothetical protein |
| 1771324 | 1770776 | FNP_1409 | probable transcriptional regulator |
| 1775226 | 1774858 | FNP_1413 | hypothetical protein |
| 1775962 | 1776174 | FNP_1415 | hypothetical protein |
| 1776944 | 1777525 | FNP_1416 | hypothetical protein |
| 1777450 | 1777355 | FNP_1417 | hypothetical protein |
| 1778158 | 1777466 | FNP_1418 | possible transcriptional regulator |
| 1778344 | 1778544 | FNP_1419 | possible transcriptional regulator |
| 1778754 | 1778825 | FNP_1420 | hypothetical protein |
| 1778825 | 1779562 | FNP_1421 | hypothetical protein |
| 1779581 | 1780033 | FNP_1422 | hypothetical protein |
| 1780051 | 1780377 | FNP_1423 | hypothetical protein |
| 1780374 | 1780478 | FNP_1424 | hypothetical protein |
| 1780468 | 1780737 | FNP_1425 | hypothetical protein |
| 1781048 | 1781344 | FNP_1426 | hypothetical protein |
| 1781348 | 1781608 | FNP_1427 | hypothetical protein |
| 1781598 | 1783643 | FNP_1428 | possible DNA replication protein |
| 1784078 | 1784671 | FNP_1429 | hypothetical protein |
| 1784702 | 1784866 | FNP_1430 | hypothetical protein |
| 1784869 | 1785930 | FNP_1431 | probable integrase |
| 1785987 | 1786106 | FNP_1432 | hypothetical protein |
| 1797256 | 1797354 | FNP_1445 | hypothetical protein |
| 1855703 | 1856740 | FNP_1496 | conserved hypothetical protein |
| 1873601 | 1874140 | FNP_1512 | hypothetical protein |
| 1875776 | 1876024 | FNP_1515 | hypothetical protein |
| 1878267 | 1878115 | FNP_1518 | conserved hypothetical protein |
| 1879501 | 1880670 | FNP_1522 | conserved hypothetical protein |
| 1881745 | 1882815 | FNP_1524 | possible DMT superfamily drug/metabolite transporter membrane protein |
| 1887763 | 1887879 | FNP_1530 | hypothetical protein |
| 1907328 | 1906972 | FNP_1546 | probable inner membrane protein |
| 1912065 | 1911796 | FNP_1552 | hypothetical protein |
| 1914831 | 1914736 | FNP_1556 | hypothetical protein |
| 1917400 | 1917032 | FNP_1558 | autoinducer AI2 synthesis protein LuxS |
| 1920501 | 1919587 | FNP_1562 | homoserine O-succinyltransferase |
| 1921180 | 1920644 | FNP_1563 | serine O-acetyltransferase |
| 1929016 | 1929498 | FNP_1572 | possible acetyltransferase |
| 1945803 | 1947365 | FNP_1590 | type I site-specific deoxyribonuclease, methyltransferase subunit |
| 1947376 | 1948806 | FNP_1591 | possible transcriptional regulator |
| 1949030 | 1950319 | FNP_1592 | type I site-specific deoxyribonuclease, specificity subunit |
| 1950327 | 1950863 | FNP_1593 | pseudogene of type I site-specific deoxyribonuclease, specificity subunit |
| 1950881 | 1953922 | FNP_1594 | type I site-specific deoxyribonuclease, restriction subunit |
| 1955466 | 1953964 | FNP_1595 | flotillin-like protein |
| 1957503 | 1957688 | FNP_1598 | hypothetical protein |
| 1957737 | 1957913 | FNP_1599 | conserved hypothetical protein |
| 1958401 | 1958024 | FNP_1600 | hypothetical protein |
| 1959540 | 1959256 | FNP_1603 | possible DNA damage-inducible protein |
| 1963649 | 1964059 | FNP_1609 | conserved hypothetical protein |
| 1964077 | 1964643 | FNP_1610 | possible integral membrane protein |
| 1964703 | 1964831 | FNP_1611 | conserved hypothetical protein |
| 1964834 | 1965013 | FNP_1612 | hypothetical protein |
| 1965023 | 1965472 | FNP_1613 | possible flavodoxin protein |
| 1965491 | 1966462 | FNP_1614 | possible esterase |
| 1966463 | 1966519 | FNP_1615 | hypothetical protein |
| 1967038 | 1966619 | FNP_1617 | possible transcriptional regulator |
| 1967786 | 1967935 | FNP_1618 | conserved hypothetical protein |
| 1967978 | 1968121 | FNP_1619 | hypothetical protein |
| 1968129 | 1968314 | FNP_1620 | hypothetical protein |
| 1968583 | 1969827 | FNP_1621 | transposase |
| 1969990 | 1970484 | FNP_1622 | conserved hypothetical protein |
| 1999399 | 1999449 | FNP_1639 | hypothetical protein |
| 2002006 | 1999829 | FNP_1640 | conserved hypothetical protein |
| 2002979 | 2001984 | FNP_1641 | probable phosphohydrolase |
| 2017273 | 2017491 | FNP_1652 | conserved hypothetical protein |
| 2017658 | 2017828 | FNP_1653 | hypothetical protein |
| 2018187 | 2018354 | FNP_1655 | hypothetical protein |
| 2025301 | 2024189 | FNP_1662 | bacteriophage integrase |
| 2025833 | 2025285 | FNP_1663 | hypothetical protein |
| 2026091 | 2026192 | FNP_1664 | hypothetical protein |
| 2026189 | 2026266 | FNP_1665 | hypothetical protein |
| 2026263 | 2026469 | FNP_1666 | hypothetical protein |
| 2026486 | 2027011 | FNP_1667 | pseudogene of bacteriophage antirepressor |
| 2027004 | 2027339 | FNP_1668 | hypothetical protein |
| 2027332 | 2027514 | FNP_1669 | hypothetical protein |
| 2027558 | 2027881 | FNP_1670 | hypothetical protein |
| 2027878 | 2029083 | FNP_1671 | SWF/SNF family helicase |
| 2029076 | 2030008 | FNP_1672 | possible bacteriophage endonuclease |
| 2030056 | 2030718 | FNP_1673 | possible prophage antirepressor |
| 2030756 | 2031412 | FNP_1674 | conserved hypothetical protein |
| 2031597 | 2032013 | FNP_1675 | hypothetical protein |
| 2032091 | 2033854 | FNP_1676 | possible DNA-directed DNA polymerase II |
| 2033885 | 2035804 | FNP_1677 | probable ATPase |
| 2036369 | 2036806 | FNP_1678 | conserved hypothetical protein |
| 2036807 | 2036989 | FNP_1679 | hypothetical protein |
| 2037013 | 2037204 | FNP_1680 | hypothetical protein |
| 2037206 | 2037310 | FNP_1681 | hypothetical protein |
| 2038001 | 2038411 | FNP_1683 | conserved hypothetical bacteriophage protein |
| 2038549 | 2039034 | FNP_1684 | probable bacteriophage terminase, small subunit |
| 2039035 | 2040759 | FNP_1685 | possible bacteriophage terminase large subunit |
| 2040756 | 2041970 | FNP_1686 | probable bacteriophage portal protein |
| 2041963 | 2042673 | FNP_1687 | endopeptidase Clp |
| 2042678 | 2043784 | FNP_1688 | bacteriophage capsid protein |
| 2043796 | 2044089 | FNP_1689 | possible bacteriophage packaging protein |
| 2044082 | 2044417 | FNP_1690 | hypothetical protein |
| 2044422 | 2044841 | FNP_1691 | hypothetical protein |
| 2044841 | 2045281 | FNP_1692 | hypothetical protein |
| 2045295 | 2046359 | FNP_1693 | bacteriophage PBSX protein XkdK |
| 2046372 | 2046803 | FNP_1694 | bacteriophage PBSX protein XkdM |
| 2046817 | 2047206 | FNP_1695 | hypothetical protein |
| 2047209 | 2047355 | FNP_1696 | hypothetical protein |
| 2047426 | 2047671 | FNP_1697 | hypothetical protein |
| 2047745 | 2050096 | FNP_1698 | conserved hypothetical bacteriophage protein |
| 2050111 | 2050656 | FNP_1699 | hypothetical protein |
| 2050666 | 2051646 | FNP_1700 | bacteriophage PBSX protein XkdQ |
| 2051639 | 2052163 | FNP_1701 | hypothetical protein |
| 2052142 | 2052594 | FNP_1702 | hypothetical protein |
| 2052591 | 2053649 | FNP_1703 | bacteriophage PBSX protein XkdT |
| 2053650 | 2054306 | FNP_1704 | conserved hypothetical protein |
| 2055064 | 2055777 | FNP_1706 | conserved hypothetical protein |
| 2057380 | 2057709 | FNP_1710 | hypothetical protein |
| 2057821 | 2058333 | FNP_1711 | hypothetical protein |
| 2058542 | 2058703 | FNP_1712 | hypothetical protein |
| 2059204 | 2060226 | FNP_1713 | hypothetical protein |
| 2060262 | 2061722 | FNP_1714 | hypothetical protein |
| 2062905 | 2061817 | FNP_1715 | conserved hypothetical protein |
| 2063814 | 2062921 | FNP_1716 | site-specific DNA-methyltransferase (adenine-specific) |
| 2072642 | 2073499 | FNP_1724 | conserved hypothetical protein |
| 2101031 | 2101123 | FNP_1754 | hypothetical protein |
| 2101123 | 2101524 | FNP_1755 | transposase-like protein A |
| 2101537 | 2102964 | FNP_1756 | conserved hypothetical protein |
| 2103828 | 2103349 | FNP_1757 | hypothetical protein |
| 2104307 | 2104077 | FNP_1759 | hypothetical protein |
| 2107600 | 2107698 | FNP_1763 | hypothetical protein |
| 2114755 | 2114663 | FNP_1766 | hypothetical protein |
| 2125957 | 2125907 | FNP_1776 | hypothetical protein |
| 2151469 | 2150135 | FNP_1794 | NADH oxidase |
| 2169262 | 2168933 | FNP_1815 | conserved hypothetical protein |
| 2170669 | 2169308 | FNP_1816 | possible ESS family glutamate:sodium (Na+) symporter |
| 2174023 | 2174136 | FNP_1820 | hypothetical protein |
| 2174309 | 2175025 | FNP_1821 | hypothetical protein |
| 2175029 | 2176399 | FNP_1822 | hypothetical protein |
| 2176396 | 2176710 | FNP_1823 | hypothetical protein |
| 2176779 | 2177429 | FNP_1824 | hypothetical protein |
| 2177447 | 2177743 | FNP_1825 | hypothetical protein |
| 2177793 | 2177512 | FNP_1826 | hypothetical protein |
| 2177756 | 2177887 | FNP_1827 | hypothetical protein |
| 2177884 | 2178345 | FNP_1828 | conserved hypothetical protein |
| 2178338 | 2178697 | FNP_1829 | hypothetical protein |
| 2178758 | 2179150 | FNP_1830 | hypothetical protein |
| 2179165 | 2179311 | FNP_1831 | hypothetical protein |
| 2179370 | 2179567 | FNP_1832 | hypothetical protein |
| 2179654 | 2181795 | FNP_1833 | topoisomerase |
| 2181810 | 2184578 | FNP_1834 | conserved hypothetical protein |
| 2184869 | 2184967 | FNP_1835 | hypothetical protein |
| 2185082 | 2186038 | FNP_1836 | hypothetical protein |
| 2186094 | 2186888 | FNP_1837 | hypothetical protein |
| 2186904 | 2187209 | FNP_1838 | hypothetical protein |
| 2188971 | 2189510 | FNP_1839 | possible protease TraF |
| 2189667 | 2189810 | FNP_1840 | hypothetical protein |
| 2189829 | 2190401 | FNP_1841 | hypothetical protein |
| 2190438 | 2190794 | FNP_1842 | hypothetical protein |
| 2190865 | 2191131 | FNP_1843 | hypothetical protein |
| 2191143 | 2192234 | FNP_1844 | possible zeta toxin |
| 2192352 | 2192513 | FNP_1845 | hypothetical protein |
| 2193729 | 2194400 | FNP_1847 | hypothetical protein |
| 2194635 | 2194543 | FNP_1848 | hypothetical protein |
| 2194721 | 2195722 | FNP_1849 | hypothetical protein |
| 2195750 | 2196142 | FNP_1850 | hypothetical protein |
| 2196255 | 2196350 | FNP_1851 | hypothetical protein |
| 2196491 | 2197396 | FNP_1852 | possible bacteriophage resistance protein |
| 2197746 | 2197456 | FNP_1853 | hypothetical protein |
| 2198333 | 2197746 | FNP_1854 | conserved hypothetical protein |
| 2199341 | 2198484 | FNP_1855 | hypothetical protein |
| 2199770 | 2199357 | FNP_1856 | hypothetical protein |
| 2200212 | 2199754 | FNP_1857 | hypothetical protein |
| 2200868 | 2200263 | FNP_1858 | possible DNA integrase |
| 2201264 | 2203675 | FNP_1859 | pseudogene of probable plasmid mobilization protein |
| 2203663 | 2204184 | FNP_1860 | hypothetical protein |
| 2204174 | 2204473 | FNP_1861 | hypothetical protein |
| 2204508 | 2204825 | FNP_1862 | hypothetical protein |
| 2204934 | 2205188 | FNP_1863 | hypothetical protein |
| 2205860 | 2206081 | FNP_1865 | hypothetical protein |
| 2206078 | 2206322 | FNP_1866 | hypothetical protein |
| 2206485 | 2206805 | FNP_1867 | hypothetical protein |
| 2206819 | 2207523 | FNP_1868 | probable conjugal transfer protein TrbF |
| 2207535 | 2208371 | FNP_1869 | probable conjugal transfer protein TrbG |
| 2208381 | 2209583 | FNP_1870 | probable conjugal transfer protein TrbI |
| 2209586 | 2211616 | FNP_1871 | probable conjugal transfer protein TraG |
| 2211762 | 2212076 | FNP_1872 | hypothetical protein |
| 2212045 | 2213010 | FNP_1873 | conjugal transfer protein TrbB |
| 2213019 | 2213282 | FNP_1874 | hypothetical protein |
| 2213293 | 2216031 | FNP_1875 | possible conjugal transfer protein TrbE |
| 2216069 | 2216251 | FNP_1876 | hypothetical protein |
| 2216275 | 2217012 | FNP_1877 | possible outer membrane protein |
| 2217119 | 2217268 | FNP_1878 | hypothetical protein |
| 2217243 | 2218775 | FNP_1879 | probable recombinase |
| 2243714 | 2243472 | FNP_1899 | conserved hypothetical protein |
| 2243771 | 2243992 | FNP_1900 | hypothetical protein |
| 2246060 | 2244600 | FNP_1902 | SSS family pantothenate:sodium (Na+) symporter |
| 2246257 | 2246060 | FNP_1903 | hypothetical protein |
| 2247821 | 2246274 | FNP_1904 | possible AraC family transcriptional regulator |
| 2248550 | 2247876 | FNP_1905 | conserved hypothetical protein |
| 2256774 | 2258819 | FNP_1913 | conserved hypothetical protein |
| 2259272 | 2259207 | FNP_1914 | hypothetical protein |
| 2261954 | 2262571 | FNP_1919 | probable transposase |
| 2262552 | 2263118 | FNP_1920 | possible transposase |
| 2278586 | 2278900 | FNP_1929 | possible transcriptional regulator |
| 2284959 | 2283769 | FNP_1935 | bifunctional pyridoxal phosphate enzyme/beta-cystathionase |
| 2286360 | 2284984 | FNP_1936 | APC family amino acid-polyamine-organocation transporter |
| 2286478 | 2287077 | FNP_1937 | probable transcriptional regulator |
| 2289261 | 2289710 | FNP_1940 | hypothetical protein |
| 2295400 | 2296584 | FNP_1946 | probable transposase |
| 2296729 | 2296589 | FNP_1947 | hypothetical protein |
| 2298529 | 2298278 | FNP_1949 | probable membrane protein |
| 2299052 | 2298558 | FNP_1950 | hypothetical protein |
| 2299473 | 2299078 | FNP_1951 | hypothetical protein |
| 2300585 | 2299671 | FNP_1952 | branched-chain-amino-acid transaminase |
| 2300703 | 2301086 | FNP_1953 | conserved hypothetical protein |
| 2305107 | 2303389 | FNP_1957 | possible ATP-binding protein |
| 2305273 | 2305193 | FNP_1958 | hypothetical protein |
| 2307525 | 2306770 | FNP_1961 | conserved hypothetical protein |
| 2317642 | 2317091 | FNP_1969 | possible flavoprotein |
| 2325228 | 2324974 | FNP_1979 | hypothetical protein |
| 2330613 | 2330539 | FNP_1985 | hypothetical protein |
| 2349742 | 2350575 | FNP_2007 | conserved hypothetical protein |
| 2384195 | 2382462 | FNP_2037 | conserved hypothetical protein |
| 2384413 | 2384186 | FNP_2038 | conserved hypothetical protein |
| 2385375 | 2384413 | FNP_2039 | conserved hypothetical protein |
| 2385721 | 2385467 | FNP_2040 | hypothetical protein |
| 2387249 | 2387097 | FNP_2044 | hypothetical protein |
| 2387724 | 2387269 | FNP_2045 | hypothetical protein |
| 2388244 | 2387744 | FNP_2046 | hypothetical protein |
| 2388428 | 2388285 | FNP_2047 | hypothetical protein |
| 2392811 | 2392443 | FNP_2050 | conserved hypothetical protein |
| 2398884 | 2398603 | FNP_2055 | pseudogene of possible metal-dependent RNase |
| 2398708 | 2399028 | FNP_2056 | pseudogene of conserved hypothetical protein |
| 2399596 | 2398838 | FNP_2057 | ATP-binding protein |
| 2400503 | 2400294 | FNP_2059 | hypothetical protein |
| 2405934 | 2407094 | FNP_2067 | probable transposase |
| 2420145 | 2420041 | FNP_2076 | hypothetical protein |

**Table S1b. FNP ORFS not in FNN**

| **Start** | **Stop** | **Locus Tag** | **Definition** |
| --- | --- | --- | --- |
| 563 | 180 | FNP_2085 | fusobacterial conserved hypothetical protein |
| 107334 | 107239 | FNP_2192 | pseudogene of fusobacterial conserved hypothetical protein |
| 107591 | 107334 | FNP_2193 | conserved hypothetical protein |
| 193660 | 193181 | FNP_2259 | conserved hypothetical protein |
| 195256 | 193685 | FNP_2260 | AAA family ATP-binding protein |
| 195708 | 195268 | FNP_2261 | fusobacterial conserved hypothetical protein |
| 195926 | 195708 | FNP_2262 | fusobacterial conserved hypothetical protein |
| 197659 | 195938 | FNP_2263 | fusobacterial conserved hypothetical protein |
| 213340 | 213528 | FNP_2277 | fusobacterial conserved hypothetical protein |
| 263164 | 262301 | FNP_2317 | fusobacterial conserved hypothetical protein |
| 295169 | 295375 | FNP_2344 | fusobacterial conserved hypothetical protein |
| 395213 | 395380 | FNP_0017 | conserved hypothetical protein |
| 395960 | 396814 | FNP_0020 | site-specific DNA-methyltransferase (adenine-specific) |
| 431036 | 431269 | FNP_0055 | fusobacterial conserved hypothetical protein |
| 474787 | 475041 | FNP_0095 | antitoxin of toxin-antitoxin system |
| 475034 | 475294 | FNP_0096 | conserved hypothetical protein |
| 482153 | 482683 | FNP_0105 | conserved hypothetical protein |
| 483589 | 484503 | FNP_0108 | fusobacterial conserved hypothetical protein |
| 486680 | 487060 | FNP_0114 | fusobacterial conserved hypothetical protein |
| 487064 | 487732 | FNP_0115 | conserved hypothetical protein |
| 519776 | 520108 | FNP_0153 | fusobacterial conserved hypothetical protein |
| 614332 | 613763 | FNP_0239 | possible transposase |
| 629326 | 630237 | FNP_0255 | conserved hypothetical protein |
| 642043 | 642405 | FNP_0270 | fusobacterial conserved hypothetical protein |
| 686557 | 686976 | FNP_0309 | flavin mononucleotide-binding protein |
| 706323 | 705715 | FNP_0329 | conserved hypothetical protein |
| 714683 | 715513 | FNP_0336 | possible dipeptide/oligopeptide/nickel (Ni2+) ABC superfamily ATP binding cassette transporter, membrane protein |
| 734198 | 733344 | FNP_0357 | LysR family transcriptional regulator |
| 734374 | 734553 | FNP_0358 | probable citrate lyase, gamma subunit |
| 734550 | 734654 | FNP_0359 | citrate (pro-3S)-lyase |
| 864743 | 866860 | FNP_0503 | glutamate--ammonia ligase |
| 900180 | 901262 | FNP_0535 | serine-pyruvate aminotransferase |
| 901259 | 901996 | FNP_0536 | cholinephosphate cytidylyltransferase |
| 910196 | 910918 | FNP_0546 | phosphoprotein phosphatase |
| 915160 | 916572 | FNP_0550 | fusobacterial conserved hypothetical protein |
| 926047 | 927675 | FNP_0564 | malate dehydrogenase |
| 927700 | 928665 | FNP_0565 | AEC family probable malonate efflux carrier |
| 930364 | 930882 | FNP_0568 | fusobacterial conserved hypothetical protein |
| 1115619 | 1114967 | FNP_0741 | pseudogene of fusobacterial conserved hypothetical protein |
| 1169100 | 1170083 | FNP_0801 | fusobacterial conserved hypothetical protein |
| 1240747 | 1241286 | FNP_0871 | fusobacterial conserved hypothetical protein |
| 1241287 | 1241988 | FNP_0872 | fusobacterial conserved hypothetical protein |
| 1257354 | 1256380 | FNP_0886 | hydrogen sulfite reductase |
| 1258173 | 1257370 | FNP_0887 | sulfite reductase (NADPH) beta subunit |
| 1259244 | 1258177 | FNP_0888 | sulfite reductase (NADPH) alpha subunit |
| 1260119 | 1259433 | FNP_0889 | Crp family transcriptional regulator |
| 1295421 | 1295299 | FNP_0933 | fusobacterial conserved hypothetical protein |
| 1298429 | 1299736 | FNP_0937 | conserved hypothetical protein |
| 1367706 | 1367293 | FNP_1018 | conserved hypothetical protein |
| 1367858 | 1368292 | FNP_1019 | MarR family transcriptional regulator |
| 1415983 | 1416378 | FNP_1075 | pseudogene of fusobacterial conserved hypothetical protein |
| 1444893 | 1443745 | FNP_1104 | UDP-N-acetylglucosamine 2-epimerase |
| 1446154 | 1444898 | FNP_1105 | possible acylneuraminate cytidylyltransferase |
| 1447822 | 1447205 | FNP_1107 | possible N-acetylneuraminate synthase |
| 1653578 | 1654801 | FNP_1300 | DNA-directed RNA polymerase sigma subunit RpoN |
| 1662788 | 1662375 | FNP_1306 | possible membrane protein |
| 1743881 | 1743162 | FNP_1384 | FIC family protein |
| 1774976 | 1775380 | FNP_1414 | bacteriophage terminase small subunit |
| 1874464 | 1875774 | FNP_1514 | conserved hypothetical protein |
| 1879351 | 1878895 | FNP_1521 | pseudogene of fusobacterial conserved hypothetical protein |
| 1902307 | 1902158 | FNP_1541 | fusobacterial conserved hypothetical protein |
| 1911803 | 1911513 | FNP_1551 | conserved hypothetical protein |
| 1959263 | 1959003 | FNP_1602 | conserved hypothetical protein |
| 1960118 | 1960633 | FNP_1605 | flavodoxin |
| 1960630 | 1961010 | FNP_1606 | MerR family transcriptional regulator |
| 1962785 | 1963630 | FNP_1608 | 2,5-didehydrogluconate reductase |
| 1998812 | 1999330 | FNP_1638 | ABC superfamily ATP binding cassette transporter, ABC protein |
| 2005138 | 2004632 | FNP_1643 | conserved hypothetical protein |
| 2011692 | 2009893 | FNP_1648 | possible ABC superfamily ATP binding cassette transporter, membrane protein |
| 2013443 | 2011704 | FNP_1649 | possible ABC superfamily ATP binding cassette transporter, membrane protein |
| 2018509 | 2019222 | FNP_1656 | N-acetylmuramoyl-L-alanine amidase |
| 2037279 | 2037746 | FNP_1682 | fusobacterial conserved hypothetical protein |
| 2054309 | 2055052 | FNP_1705 | fusobacterial conserved hypothetical protein |
| 2056091 | 2056639 | FNP_1707 | N-acetylmuramoyl-L-alanine amidase |
| 2056642 | 2056929 | FNP_1708 | fusobacterial conserved hypothetical protein |
| 2067767 | 2068327 | FNP_1719 | conserved hypothetical protein |
| 2192989 | 2193714 | FNP_1846 | ParA family chromosome partitioning ATP-binding protein |
| 2205302 | 2205547 | FNP_1864 | fusobacterial conserved hypothetical protein |
| 2301486 | 2302259 | FNP_1954 | glutamate racemase |
| 2317802 | 2318149 | FNP_1970 | fusobacterial conserved hypothetical protein |
| 2327491 | 2326907 | FNP_1981 | fusobacterial conserved hypothetical protein |
| 2328107 | 2327511 | FNP_1982 | fusobacterial conserved hypothetical protein |
| 2329503 | 2328094 | FNP_1983 | conserved hypothetical protein |
| 2334056 | 2333256 | FNP_1989 | conserved hypothetical protein, possible kinase |
| 2364604 | 2364404 | FNP_2025 | fusobacterial conserved hypothetical protein |
| 2364994 | 2364644 | FNP_2026 | fusobacterial conserved hypothetical protein |
| 2366286 | 2365276 | FNP_2027 | pseudogene of fusobacterial conserved hypothetical protein |
| 2366584 | 2366357 | FNP_2028 | fusobacterial conserved hypothetical protein |
| 2367131 | 2369287 | FNP_2029 | site-specific DNA-methyltransferase (adenine-specific) |
| 2369284 | 2372400 | FNP_2030 | probable DNA helicase |
| 2373634 | 2372447 | FNP_2031 | conserved hypothetical protein |
| 2375919 | 2373643 | FNP_2032 | conserved hypothetical protein |
| 2377034 | 2375934 | FNP_2033 | possible MoxR family ATP-binding protein |
| 2378936 | 2377038 | FNP_2034 | fusobacterial conserved hypothetical protein |
| 2380897 | 2378987 | FNP_2035 | fusobacterial conserved hypothetical protein |
| 2382359 | 2380911 | FNP_2036 | conserved hypothetical protein |

**Table S1c. FNP ORFS not found in FNV**

| **Start** | **Stop** | **Locus Tag** | **Definition** |
| --- | --- | --- | --- |
| 34635 | 35063 | FNP_2119 | possible carbon dioxide concentrating/carboxysome shell protein |
| 35082 | 35360 | FNP_2120 | possible carbon dioxide concentrating/carboxysome shell protein |
| 36734 | 37003 | FNP_2123 | possible carbon dioxide concentrating/carboxysome shell protein |
| 39537 | 40268 | FNP_2126 | major intrinsic protein (MIP) family glycerol facilitator protein |
| 76032 | 77615 | FNP_2159 | CPA1 family sodium sodium (Na+):proton (H+) antiporter |
| 92053 | 90605 | FNP_2173 | fusobacterial conserved hypothetical protein |
| 92389 | 92057 | FNP_2174 | conserved hypothetical protein |
| 93177 | 92392 | FNP_2175 | beta-lactamase |
| 94366 | 93185 | FNP_2176 | conserved hypothetical protein |
| 95478 | 94387 | FNP_2177 | L-alanine-DL-glutamate epimerase |
| 96386 | 96604 | FNP_2180 | fusobacterial conserved hypothetical protein |
| 96626 | 97285 | FNP_2181 | probable transcriptional regulator |
| 100678 | 100094 | FNP_2184 | NADH dehydrogenase (ubiquinone), RnfA subunit |
| 151815 | 150850 | FNP_2222 | site-specific DNA-methyltransferase (adenine-specific) |
| 187436 | 187062 | FNP_2253 | fusobacterial conserved hypothetical protein |
| 205992 | 205441 | FNP_2271 | conserved hypothetical protein |
| 206999 | 206100 | FNP_2272 | probable transcriptional regulator |
| 207656 | 207279 | FNP_2273 | endoribonuclease |
| 217440 | 218564 | FNP_2281 | conserved hypothetical protein |
| 230435 | 229044 | FNP_2286 | possible MFS family major facilitator transporter |
| 230651 | 231502 | FNP_2287 | RpiR family transcriptional regulator |
| 250934 | 251482 | FNP_2306 | possible biotin synthase |
| 251492 | 252286 | FNP_2307 | ABC superfamily ATP binding cassette transporter, ABC protein |
| 277578 | 277048 | FNP_2329 | conserved hypothetical protein |
| 278644 | 277571 | FNP_2330 | N utilization substance protein A |
| 279141 | 278671 | FNP_2331 | conserved hypothetical protein |
| 279590 | 280153 | FNP_2333 | bifunctional diaminohydroxyphosphoribosylaminopyrimidine deaminase/5-amino-6-(5-phosphoribosylamino)uracil reductase RibD |
| 298945 | 298793 | FNP_2351 | ribosomal protein L33 |
| 300274 | 299846 | FNP_2353 | ferric uptake regulator |
| 300832 | 300377 | FNP_2354 | possible acetyltransferase |
| 305086 | 303899 | FNP_2358 | DAACS family dicarboxylate/amino acid:cation (Na+ or H+) symporter |
| 323228 | 321873 | FNP_2372 | AGCS family alanine (or glycine):cation symporter carrier |
| 331243 | 330467 | FNP_2379 | ABC superfamily ATP binding cassette transporter, ABC protein |
| 332091 | 331246 | FNP_2380 | ABC superfamily ATP binding cassette transporter, membrane protein |
| 333279 | 332350 | FNP_2382 | ABC superfamily ATP binding cassette transporter, binding protein |
| 371994 | 371434 | FNP_2424 | conserved hypothetical protein |
| 402922 | 403989 | FNP_0031 | fusobacterial conserved hypothetical protein |
| 422383 | 422571 | FNP_0042 | fusobacterial conserved hypothetical protein |
| 434739 | 435950 | FNP_0060 | threonine dehydratase |
| 447238 | 452388 | FNP_0072 | conserved hypothetical protein |
| 465278 | 464979 | FNP_0088 | S4 domain RNA-binding protein |
| 468735 | 469211 | FNP_0090 | fusobacterial conserved hypothetical protein |
| 469457 | 471178 | FNP_0091 | succinate dehydrogenase flavoprotein subunit |
| 475304 | 475807 | FNP_0096 | ribosomal-protein-alanine N-acetyltransferase |
| 476397 | 477047 | FNP_0099 | DNA-(apurinic or apyrimidinic site) lyase |
| 477126 | 478322 | FNP_0100 | cysteine sulfinate desulfinase |
| 478385 | 478771 | FNP_0101 | NifU protein |
| 478854 | 480134 | FNP_0102 | M01 family membrane alanyl aminopeptidase |
| 480171 | 481661 | FNP_0103 | M32 family carboxypeptidase |
| 501700 | 502068 | FNP_0126 | ethanolamine utilization protein EutS |
| 502071 | 502508 | FNP_0127 | ethanolamine utilization protein EutP |
| 502498 | 503076 | FNP_0128 | ethanolamine response regulator |
| 503076 | 504464 | FNP_0129 | ethanolamine sensor histidine kinase |
| 504578 | 506008 | FNP_0131 | ethanolamine utilization protein EutA |
| 506035 | 507402 | FNP_0132 | ethanolamine ammonia-lyase, large subunit EutB |
| 507414 | 508301 | FNP_0133 | ethanolamine ammonia-lyase, small subunit EutC |
| 508505 | 509158 | FNP_0134 | ethanolamine utilization protein EutL |
| 509169 | 509615 | FNP_0135 | ethanolamine utilization protein EutM |
| 509650 | 509934 | FNP_0136 | possible carbon dioxide concentrating/carboxysome shell protein |
| 510043 | 511491 | FNP_0137 | aldehyde dehydrogenase (NAD+) |
| 511705 | 512472 | FNP_0138 | cob(I)yrinic acid a,c-diamide adenosyltransferase |
| 512674 | 513261 | FNP_0139 | fusobacterial conserved hypothetical protein |
| 513263 | 513511 | FNP_0140 | ethanolamine utilization protein EutN |
| 513524 | 513859 | FNP_0141 | fusobacterial conserved hypothetical protein |
| 513859 | 514941 | FNP_0142 | ethanolamine utilization protein EutH |
| 514952 | 515401 | FNP_0143 | ethanolamine utilization protein EutQ |
| 530403 | 530813 | FNP_0157 | fusobacterial conserved hypothetical protein |
| 530833 | 531228 | FNP_0158 | conserved hypothetical protein |
| 531328 | 532326 | FNP_0159 | possible hemolysin |
| 536803 | 538044 | FNP_0165 | 3-oxoacyl-[acyl-carrier-protein] synthase |
| 613050 | 613271 | FNP_0236 | fusobacterial conserved hypothetical protein |
| 613276 | 613515 | FNP_0237 | possible toxin translational regulator |
| 646712 | 646906 | FNP_0275 | MerTP family mercury (Hg2+) permease, binding protein MerP |
| 650213 | 651133 | FNP_0278 | fusobacterial conserved hypothetical protein |
| 672022 | 672705 | FNP_0293 | fusobacterial conserved hypothetical protein |
| 672810 | 673199 | FNP_0294 | fusobacterial conserved hypothetical protein |
| 673353 | 674288 | FNP_0295 | conserved hypothetical protein |
| 677964 | 678734 | FNP_0299 | probable integrase/recombinase |
| 679629 | 681056 | FNP_0301 | possible transcriptional regulator |
| 683318 | 684073 | FNP_0305 | ABC superfamily ATP binding cassette transporter, ABC protein |
| 716593 | 717216 | FNP_0339 | possible cobalamin/iron (Fe3+)-siderophore ABC superfamily ATP binding cassette transporter, ABC protein |
| 729591 | 729085 | FNP_0351 | fusobacterial conserved hypothetical protein |
| 730856 | 729669 | FNP_0352 | tryptophan synthase beta chain |
| 745817 | 744471 | FNP_0371 | AGCS family alanine (or glycine):sodium (Na+ ) symporter carrier AlsT |
| 753531 | 753211 | FNP_0381 | fusobacterial conserved hypothetical protein |
| 753690 | 754145 | FNP_0382 | fusobacterial conserved hypothetical protein |
| 755507 | 754197 | FNP_0383 | conserved hypothetical transmembrane protein |
| 763373 | 763828 | FNP_0392 | D-tyrosyl-tRNA(Tyr) deacylase |
| 765149 | 766528 | FNP_0395 | NhaC family sodium:proton (Na+:H+) antiporter |
| 793511 | 794572 | FNP_0426 | iron (Fe3+) ABC superfamily ATP binding cassette transporter, binding protein |
| 795363 | 796424 | FNP_0428 | iron (Fe3+) ABC superfamily ATP binding cassette transporter, binding protein |
| 804352 | 805092 | FNP_0436 | outer membrane protein |
| 838127 | 838516 | FNP_0471 | biopolymer transport protein ExbD |
| 843986 | 844450 | FNP_0478 | single-stranded DNA binding protein |
| 860117 | 859948 | FNP_0494 | fusobacterial conserved hypothetical protein |
| 860664 | 861020 | FNP_0497 | ribosomal protein S13 |
| 861066 | 861455 | FNP_0498 | ribosomal protein S11 |
| 896606 | 895221 | FNP_0531 | OFeT family oxidase-dependent iron (Fe2+) transporter |
| 903907 | 904767 | FNP_0539 | possible glycosyltransferase |
| 904782 | 905864 | FNP_0540 | possible glycosyltransferase |
| 905879 | 906610 | FNP_0541 | probable glycosyltransferase |
| 907344 | 907988 | FNP_0543 | fusobacterial conserved hypothetical protein |
| 908004 | 909200 | FNP_0544 | possible O-antigen ligase |
| 911633 | 910926 | FNP_0547 | fusobacterial conserved hypothetical protein |
| 920500 | 921171 | FNP_0557 | fusobacterial conserved hypothetical protein |
| 954182 | 953910 | FNP_0593 | fusobacterial conserved hypothetical protein |
| 962784 | 963545 | FNP_0602 | pseudogene of nickel (Ni2+) ABC superfamily ATP binding cassette transporter, membrane protein |
| 967296 | 968195 | FNP_0608 | conserved hypothetical protein |
| 968224 | 968976 | FNP_0609 | conserved hypothetical protein |
| 968966 | 970510 | FNP_0610 | conserved hypothetical protein |
| 970523 | 971425 | FNP_0611 | conserved hypothetical protein |
| 971439 | 972539 | FNP_0612 | conserved hypothetical protein |
| 972631 | 975069 | FNP_0613 | ATP-dependent helicase |
| 975139 | 975633 | FNP_0614 | possible RecB family recombination protein |
| 975645 | 976637 | FNP_0615 | conserved hypothetical protein |
| 976642 | 976920 | FNP_0616 | conserved hypothetical protein |
| 999787 | 996517 | FNP_0631 | probable ATP-dependent helicase |
| 1018081 | 1019364 | FNP_0644 | conserved hypothetical protein |
| 1023429 | 1022605 | FNP_0649 | glucosamine-6-phosphate deaminase |
| 1024594 | 1025364 | FNP_0651 | FNT family formate-nitrite transporter |
| 1032696 | 1031953 | FNP_0656 | phosphate/phosphonate ABC superfamily ATP binding cassette transporter, ABC protein |
| 1034373 | 1035533 | FNP_0659 | N-acetylglucosamine-6-phosphate deacetylase |
| 1061885 | 1062664 | FNP_0684 | dipeptide/oligopeptide/nickel (Ni2+) ABC superfamily ATP binding cassette transporter, ABC protein |
| 1064167 | 1063679 | FNP_0686 | possible NTP pyrophosphohydrolase |
| 1064658 | 1064191 | FNP_0687 | possible GNAT family acetyltransferase |
| 1071858 | 1070512 | FNP_0693 | possible AAA+ superfamily ATPase |
| 1093038 | 1091344 | FNP_0716 | ABC superfamily ATP binding cassette transporter, binding protein |
| 1093600 | 1093166 | FNP_0717 | possible bacterioferrin |
| 1094606 | 1094857 | FNP_0719 | conserved hypothetical protein |
| 1113070 | 1112300 | FNP_0738 | fusobacterial conserved hypothetical protein |
| 1116815 | 1116048 | FNP_0743 | fusobacterial conserved hypothetical protein |
| 1117586 | 1116816 | FNP_0744 | fusobacterial conserved hypothetical protein |
| 1120072 | 1119746 | FNP_0749 | fusobacterial conserved hypothetical protein |
| 1134874 | 1134161 | FNP_0766 | probable LIV-E family branched-chain amino acid exporter |
| 1137188 | 1136280 | FNP_0769 | DMT superfamily drug/metabolite transporter |
| 1145279 | 1144683 | FNP_0777 | TetR family transcriptional regulator |
| 1152175 | 1153044 | FNP_0785 | pseudouridylate synthase RluA |
| 1153081 | 1154373 | FNP_0786 | NCS2 family nucleobase:cation symporter-2 |
| 1154516 | 1154824 | FNP_0787 | histone-binding protein |
| 1159987 | 1159148 | FNP_0791 | 3-hydroxybutyryl-CoA dehydrogenase |
| 1197263 | 1197913 | FNP_0826 | conserved hypothetical protein |
| 1238630 | 1239310 | FNP_0868 | fusobacterial conserved hypothetical protein |
| 1271489 | 1270764 | FNP_0901 | fusobacterial conserved hypothetical protein |
| 1275297 | 1276244 | FNP_0907 | fusobacterial conserved hypothetical protein |
| 1301403 | 1301786 | FNP_0939 | fusobacterial conserved hypothetical protein |
| 1330393 | 1329281 | FNP_0972 | FomP family fusobacterial outer membrane porin FomA |
| 1345846 | 1345550 | FNP_0989 | fusobacterial conserved hypothetical protein |
| 1347852 | 1347250 | FNP_0993 | possible C56 family protease |
| 1350172 | 1349993 | FNP_0998 | fusobacterial conserved hypothetical protein |
| 1406442 | 1405948 | FNP_1054 | ribosomal protein S5 |
| 1406834 | 1406466 | FNP_1055 | ribosomal protein L18 |
| 1408133 | 1407846 | FNP_1058 | ribosomal protein S14 |
| 1414636 | 1414325 | FNP_1072 | ribosomal protein S10 |
| 1414856 | 1415302 | FNP_1073 | fusobacterial conserved hypothetical protein |
| 1430870 | 1429668 | FNP_1090 | dTDP-glucose 4,6-dehydratase |
| 1452549 | 1451707 | FNP_1111 | probable glycosyltransferase |
| 1454376 | 1453789 | FNP_1113 | probable glycosyltransferase |
| 1456203 | 1454380 | FNP_1114 | possible nucleoside-diphosphate sugar epimerase |
| 1457201 | 1456203 | FNP_1115 | fusobacterial conserved hypothetical protein |
| 1502725 | 1505367 | FNP_1154 | Sec family Type II general secretory pathway protein SecA |
| 1542297 | 1541551 | FNP_1188 | conserved hypothetical protein |
| 1574272 | 1574925 | FNP_1226 | probable transcriptional regulator |
| 1576430 | 1577626 | FNP_1228 | conserved hypothetical protein |
| 1577736 | 1578602 | FNP_1229 | conserved hypothetical protein |
| 1601074 | 1600970 | FNP_1247 | pseudogene of hemolysin activator protein |
| 1610407 | 1609937 | FNP_1255 | flavodoxin |
| 1623321 | 1622707 | FNP_1278 | conserved hypothetical protein |
| 1624282 | 1623389 | FNP_1268 | possible transcriptional regulator |
| 1642616 | 1643812 | FNP_1287 | 3-phosphoglycerate kinase |
| 1683024 | 1682248 | FNP_1326 | ABC superfamily ATP binding cassette transporter, ABC protein |
| 1686924 | 1685305 | FNP_1329 | chaperone GroEL |
| 1687214 | 1686942 | FNP_1330 | chaperone GroES |
| 1697991 | 1697698 | FNP_1341 | possible membrane protein |
| 1698130 | 1699533 | FNP_1342 | fusobacterial conserved hypothetical protein |
| 1741366 | 1740875 | FNP_1380 | conserved hypothetical protein |
| 1750650 | 1752584 | FNP_1391 | possible autotransporter adhesin |
| 1800447 | 1800857 | FNP_1450 | MscL family large conductance mechanosensitive ion channel |
| 1815940 | 1815449 | FNP_1464 | conserved hypothetical protein |
| 1833896 | 1828839 | FNP_1476 | conserved hypothetical protein |
| 1834318 | 1833905 | FNP_1477 | conserved hypothetical protein |
| 1843430 | 1842702 | FNP_1483 | amino acid ABC superfamily ATP binding cassette transporter, ABC protein |
| 1867786 | 1868250 | FNP_1507 | fusobacterial conserved hypothetical protein |
| 1877284 | 1878069 | FNP_1517 | fusobacterial conserved hypothetical protein |
| 1878634 | 1878245 | FNP_1519 | conserved hypothetical protein |
| 1878809 | 1878609 | FNP_1520 | fusobacterial conserved hypothetical protein |
| 1934004 | 1934936 | FNP_1578 | methionyl-tRNA formyltransferase |
| 1958977 | 1958426 | FNP_1601 | fusobacterial conserved hypothetical protein |
| 1967038 | 1966619 | FNP_1616 | conserved hypothetical protein |
| 2009558 | 2009815 | FNP_1647 | ribosomal protein L28 |
| 2017830 | 2018186 | FNP_1651 | pseudogene of ABC superfamily ATP binding cassette transporter, binding protein |
| 2072211 | 2072651 | FNP_1723 | fusobacterial conserved hypothetical protein |
| 2092425 | 2092733 | FNP_1744 | fusobacterial conserved hypothetical protein |
| 2092750 | 2093148 | FNP_1745 | fusobacterial conserved hypothetical protein |
| 2093267 | 2093512 | FNP_1746 | ribosomal protein L31 |
| 2104057 | 2103905 | FNP_1758 | fusobacterial conserved hypothetical protein |
| 2105256 | 2104327 | FNP_1760 | possible transcriptional regulator |
| 2106322 | 2107530 | FNP_1762 | immunosuppressive protein FipA |
| 2125759 | 2125959 | FNP_1775 | fusobacterial conserved hypothetical protein |
| 2130357 | 2130797 | FNP_1781 | fusobacterial conserved hypothetical protein |
| 2151892 | 2152107 | FNP_1795 | cold shock protein CspC |
| 2153253 | 2152162 | FNP_1796 | possible ammonia monooxygenase |
| 2154280 | 2153240 | FNP_1797 | conserved hypothetical protein |
| 2154439 | 2154975 | FNP_1798 | conserved hypothetical protein |
| 2172070 | 2173269 | FNP_1818 | conserved hypothetical protein |
| 2206078 | 2206322 | FNP_1866 | pseudogene of possible plasmid addiction protein |
| 2226995 | 2228584 | FNP_1888 | von Willebrand factor domain protein |
| 2265441 | 2265886 | FNP_1922 | tmRNA binding protein SmpB |
| 2283525 | 2283668 | FNP_1934 | fusobacterial conserved hypothetical protein |
| 2388924 | 2388454 | FNP_1938 | fusobacterial conserved hypothetical protein |
| 2402562 | 2402251 | FNP_2062 | conserved hypothetical protein |
| 2402926 | 2402627 | FNP_2063 | fusobacterial conserved hypothetical protein |
| 2403083 | 2403223 | FNP_2064 | pseudogene of fusobacterial conserved hypothetical protein |
| 2419620 | 2419877 | FNP_2075 | fusobacterial conserved hypothetical protein |
