# Supplementary material for: Genome Sequence of Fusobacterium nucleatum Subspecies Polymorphum — a Genetically Tractable Fusobacterium
Source: PLoS One. 2007 Aug 1;2(8):e659. doi: 10.1371/journal.pone.0000659 (PMC1924603; doi:10.1371/journal.pone.0000659)
Supplement: Table S2 — Clusters of ORFs unique to FNP and not in FNN or FNV. (0.74 MB DOC) [file pone.0000659.s002.doc]

Table S2. Clusters of ORFs unique to FNP and not in FNN or FNV

| **Cluster** | **# of Genes per cluster** | **FNP #** | **Start** | **Stop** | **Definition** | **Top hit in** | **e value** |
| --- | --- | --- | --- | --- | --- | --- | --- |
| I | 5 | 2115 | 31183 | 31863 | propanediol dehydratase, medium subunit | *Clostridium perfringens* | e-59 |
|  |  | 2116 | 31877 | 32386 | propanediol dehydratase, small subunit | *Clostridium perfringens* | e-36 |
|  |  | 2117 | 32409 | 34223 | propanediol dehydratase, large subunit | *Clostridium perfringens* | 0 |
|  |  | 2118 | 34223 | 34600 | conserved hypothetical protein | *Clostridium perfringens* | e-13 |
|  |  | 2119 | 34635 | 35063 | possible CO_2_ concentrating carboxysome shell protein | *Escherichia coli* | e-13 |
|  |  |  |  |  |  |  |  |
| II | 9 | 2207 | 128759 | 125970 | conserved hypothetical protein | *F. nucleatum* PK1594 | e-104 |
|  |  | 2208 | 131810 | 128760 | conserved hypothetical protein | *F. nucleatum* PK1594 | 0 |
|  |  | 2209 | 135156 | 131824 | conserved hypothetical protein | *F. nucleatum* PK1594 | 0 |
|  |  | 2210 | 138609 | 135166 | conserved hypothetical protein | *F. nucleatum* PK1594 | 0 |
|  |  | 2211 | 139951 | 138641 | hypothetical protein | none |  |
|  |  | 2212 | 140538 | 140002 | hypothetical protein | none |  |
|  |  | 2213 | 141164 | 140556 | hypothetical protein | none |  |
|  |  | 2214 | 141827 | 141180 | hypothetical protein | none |  |
|  |  | 2215 | 145221 | 141850 | conserved hypothetical protein | *Bacillus cereus* | e-81 |
|  |  |  |  |  |  |  |  |
| III | 7 | 0045 | 424671 | 425297 | hypothetical protein | none |  |
|  |  | 0046 | 425594 | 425968 | hypothetical protein | none |  |
|  |  | 0047 | 426382 | 426723 | hypothetical protein | none |  |
|  |  | 0048 | 426749 | 427312 | hypothetical protein | none |  |
|  |  | 0049 | 427349 | 428290 | hypothetical protein | none |  |
|  |  | 0050 | 428334 | 428744 | hypothetical protein | none |  |
|  |  | 0051 | 428961 | 429293 | hypothetical protein | none |  |
|  |  |  |  |  |  |  |  |
| IV | 7 | 0061 | 435961 | 437682 | acetolactate synthase large subunit | *Methanococcoides burtonii* | e-176 |
|  |  | 0062 | 437675 | 438163 | acetolactate synthase small subunit | *Methanosarcina mazei* | e-31 |
|  |  | 0063 | 438406 | 439914 | 2-isopropylmalate synthase | *Geobacter metallireducens* | e-134 |
|  |  | 0064 | 439925 | 441316 | 3-isopropylmalate dehydratase large subunit | *Listeria monocytogenes* | 0 |
|  |  | 0065 | 441316 | 441891 | 3-isopropylmalate dehydratase small subunit | *Bacillus cereus* | e-60 |
|  |  | 0066 | 442032 | 442148 | hypothetical protein | none |  |
|  |  | 0067 | 442203 | 443261 | isocitrate/isopropylmalate dehydrogenase | *Pseudomonas syringae* | e-118 |
|  |  |  |  |  |  |  |  |
| V | 5 | 0109 | 484507 | 484902 | hypothetical protein | none |  |
|  |  | 0110 | 484899 | 485051 | hypothetical protein | none |  |
|  |  | 0111 | 485061 | 485765 | hypothetical protein | none |  |
|  |  | 0112 | 485810 | 486151 | hypothetical protein | none |  |
|  |  | 0113 | 486167 | 486670 | hypothetical protein | none |  |
|  |  |  |  |  |  |  |  |
| VI | 6 | 0263 | 636577 | 636828 | hypothetical protein | none |  |
|  |  | 0264 | 636977 | 637123 | hypothetical protein | none |  |
|  |  | 0265 | 637175 | 637675 | conserved hypothetical protein | *Streptococcus mutans* | e-19 |
|  |  | 0266 | 638040 | 638690 | hypothetical protein | none |  |
|  |  | 0267 | 638758 | 639483 | hypothetical protein | none |  |
|  |  | 0268 | 639501 | 640205 | hypothetical protein | none |  |
|  |  |  |  |  |  |  |  |
| VII | 5 | 0397 | 768143 | 768844 | hypothetical protein | none |  |
|  |  | 0398 | 769162 | 769899 | hypothetical protein | none |  |
|  |  | 0399 | 770062 | 770787 | hypothetical protein | none |  |
|  |  | 0400 | 770914 | 771693 | conserved hypothetical protein | *Streptococcus mutans* | e-11 |
|  |  | 0401 | 771727 | 772839 | hypothetical protein | none |  |
|  |  |  |  |  |  |  |  |
| VIII | 7 | 0694 | 1072096 | 1072305 | hypothetical protein | none |  |
|  |  | 0695 | 1072306 | 1072353 | hypothetical protein | none |  |
|  |  | 0696 | 1072660 | 1073058 | hypothetical protein | none |  |
|  |  | 0697 | 1073177 | 1073860 | hypothetical protein | none |  |
|  |  | 0698 | 1073847 | 1074467 | hypothetical protein | none |  |
|  |  | 0699 | 1074684 | 1075682 | hypothetical protein | none |  |
|  |  | 0700 | 1075651 | 1075965 | conserved hypothetical protein | *Helicobacter pylori* | e-10 |
|  |  |  |  |  |  |  |  |
| IX | 5 | 0770 | 1137526 | 1139106 | possible ferredoxin-nitrite reductase | *Clostridium acetobutylicum* | e-62 |
|  |  | 0771 | 1139107 | 1140915 | conserved hypothetical protein | *Bacillus cereus* | e-176 |
|  |  | 0772 | 1140929 | 1141681 | possible nitrate/sulfonate/bicarbonate ABC transporter, membrane protein | *Bordetella bronchoseptica* | e-42 |
|  |  | 0773 | 1141694 | 1142455 | possible nitrate/sulfonate/bicarbonate ABC transporter, ABC protein | Synthetic construct | e-58 |
|  |  | 0774 | 1142477 | 1143514 | possible nitrate/sulfonate/bicarbonate ABC superfamily ATP binding cassette transporter, binding protein | *Pseudomonas putida* | e-22 |
|  |  |  |  |  |  |  |  |
| X | 7 | 0812 | 1180625 | 1181350 | possible zinc (Zn2+)-dependent hydrolase/metallo-beta-lactamase | *Methanosarcina barkeri* | e-15 |
|  |  | 0813 | 1181343 | 1182248 | hypothetical protein | none |  |
|  |  | 0814 | 1182264 | 1182962 | conserved hypothetical protein | *Nostoc punctiforme* | e-30 |
|  |  | 0815 | 1182977 | 1184524 | conserved hypothetical protein | *Burkholderia cepacia* | e-60 |
|  |  | 0816 | 1184521 | 1185849 | possible iron-sulfur (Fe-S) oxidoreductase | *Moorella thermoacetica* | e-93 |
|  |  | 0817 | 1185892 | 1187331 | APC family amino acid transporter | *Bacteroides thetaiotaomicron* | e-86 |
|  |  | 0818 | 1187351 | 1188730 | glutamate decarboxylase | *Bacillus cereus* | e-164 |
|  |  |  |  |  |  |  |  |
| XI | 6 | 0832 | 1202195 | 1204258 | hypothetical protein | none |  |
|  |  | 0833 | 1204264 | 1205493 | hypothetical protein | none |  |
|  |  | 0834 | 1205722 | 1205793 | hypothetical protein | none |  |
|  |  | 0835 | 1205802 | 1206173 | hypothetical protein | none |  |
|  |  | 0836 | 1206145 | 1206420 | hypothetical protein | none |  |
|  |  | 0837 | 1206356 | 1206466 | hypothetical protein | none |  |
|  |  |  |  |  |  |  |  |
| XII | 5 | 0922 | 1287021 | 1287746 | probable ABC superfamily ATP binding cassette transporter, ABC protein | *Desulfitobacterium hafniense* | e-42 |
|  |  | 0923 | 1287671 | 1288453 | probable nitrate/sulfonate/bicarbonate ABC superfamily ATP binding cassette transporter, membrane protein | *Desulfitobacterium hafniense* | e-29 |
|  |  | 0924 | 1288428 | 1289378 | probable nitrate/sulfonate/bicarbonate ABC superfamily ATP binding cassette transporter, binding protein | *Desulfitobacterium hafniense* | e-84 |
|  |  | 0925 | 1289705 | 1289899 | hypothetical protein | none |  |
|  |  | 0926 | 1289778 | 1289990 | hypothetical protein | none |  |
|  |  |  |  |  |  |  |  |
| XIII | 8 | 1096 | 1436046 | 1435852 | hypothetical protein | none |  |
|  |  | 1097 | 1436149 | 1436033 | hypothetical protein | none |  |
|  |  | 1098 | 1436693 | 1436271 | hypothetical protein | none |  |
|  |  | 1099 | 1439611 | 1436696 | hypothetical protein | none |  |
|  |  | 1100 | 1439630 | 1440187 | possible phosphatase | *Rubrobacter xylanophilus* | e-15 |
|  |  | 1101 | 1440174 | 1442105 | conserved hypothetical protein | *Pyrococcus furiosus* | e-6 |
|  |  | 1102 | 1442115 | 1442858 | possible pyrophosphorylase | *Magnetospirillum magnetotacticum* | e-16 |
|  |  | 1103 | 1442839 | 1443645 | probable glycosyltransferase | *Saccharophagus degradans* | e-66 |
|  |  |  |  |  |  |  |  |
| XIV | 17 | 1129 | 1470823 | 1472529 | conserved hypothetical protein | *Ralstonia metallidurans* | e-17 |
|  |  | 1130 | 1472638 | 1473918 | hypothetical protein | *none* |  |
|  |  | 1131 | 1473923 | 1475032 | possible AAA family ATPase | *Lactococcus lactis* | e-72 |
|  |  | 1132 | 1475748 | 1479764 | possible helicase | *Trichodesmium erythraeum* | e-53 |
|  |  | 1133 | 1479761 | 1482217 | possible ATP-binding protein | none |  |
|  |  | 1134 | 1482227 | 1483357 | hypothetical protein | none |  |
|  |  | 1135 | 1483357 | 1485207 | possible ClpA family chaperone | *Pseudomonas aeruginosa* | e-78 |
|  |  | 1136 | 1485216 | 1485782 | [formate-C-acetyltransferase]-activating enzyme | *Methano-thermobacter* | e-25 |
|  |  | 1137 | 1486017 | 1486100 | hypothetical protein | none |  |
|  |  | 1138 | 1486167 | 1487249 | hypothetical protein | none |  |
|  |  | 1139 | 1487246 | 1488007 | possible Fe-S possible pyruvate-formate lyase-activating enzyme | *Moorella thermoacetica* | e-8 |
|  |  | 1140 | 1488019 | 1489089 | possible Fe-S oxidoreductase | *Bacillus subtilis* | e-14 |
|  |  | 1141 | 1489067 | 1490296 | possible methyltransferase/Fe-S oxidoreductase | *Pyrococcus* | e-12 |
|  |  | 1142 | 1490281 | 1491795 | possible thiamine pyrophosphate enzyme | *Thermotoga maritima* | e-20 |
|  |  | 1143 | 1491806 | 1492303 | adenylyl-sulfate kinase | *Helicobacter hepaticus* | e-28 |
|  |  | 1144 | 1492307 | 1493059 | methyltransferase | *Pseudomonas fluorescens* | e-75 |
|  |  | 1145 | 1493064 | 1495379 | possible phopshoenolpyruvate synthase/pyruvate phosphate dikinase | *Campylobacter jejuni* | e-164 |
|  |  |  |  |  |  |  |  |
| XV | 8 | 1238 | 1585647 | 1586066 | hypothetical protein | none |  |
|  |  | 1239 | 1586096 | 158656 | conserved hypothetical protein | *Pasteurella multocida* | e-12 |
|  |  | 1240 | 1586683 | 1586955 | hypothetical protein | none |  |
|  |  | 1241 | 1586939 | 1587253 | hypothetical protein | none |  |
|  |  | 1242 | 1587333 | 1587728 | hypothetical protein | none |  |
|  |  | 1243 | 1588474 | 1587752 | conserved hypothetical protein | FNV | e-20 |
|  |  | 1244 | 1588957 | 1588622 | hypothetical protein |  |  |
|  |  | 1245 | 1589869 | 1589240 | hypothetical protein |  |  |
|  |  |  |  |  |  |  |  |
| XVI | 9 | 1312 | 1668533 | 1670029 | type I site-specific deoxyribonuclease, methyltransferase | *Streptococcus pneumoniae TIGR4* | e-149 |
|  |  | 1313 | 1670016 | 1671179 | type I site-specific deoxyribonuclease, specificity subunit | *Methanosarcina*  *acetivorans* | e-44 |
|  |  | 1314 | 1671176 | 1672312 | type I site-specific deoxyribonuclease, restriction subunit | *Escherichia coli* | e-22 |
|  |  | 1315 | 1672401 | 1672628 | hypothetical protein | none |  |
|  |  | 1316 | 1672601 | 1672717 | hypothetical protein | none |  |
|  |  | 1317 | 1672874 | 1672954 | hypothetical protein | none |  |
|  |  | 1318 | 1673146 | 1674393 | possible transposase | *Alicyclobacillus acidocaldarius* | e-99 |
|  |  | 1319 | 1674459 | 1674605 | hypothetical protein | none |  |
|  |  | 1320 | 1674590 | 1674901 | conserved hypothetical protein | *Listeria innocua* | e-18 |
|  |  |  |  |  |  |  |  |
| XVII | 6 | 1394 | 1753891 | 1754118 | hypothetical protein | none |  |
|  |  | 1395 | 1754185 | 1755276 | aspartate-semialdehyde dehydrogenase | *Bifidobacterium longum* | e-120 |
|  |  | 1396 | 1755257 | 1756141 | homoserine kinase ThrB | *Clostridium tetani* | e-51 |
|  |  | 1397 | 1756129 | 1757586 | threonine synthase | *Bifidobacterium longum* | e-129 |
|  |  | 1398 | 1757682 | 1758812 | homoserine dehydrogenase | *Synechocystis sp.* | e-54 |
|  |  | 1399 | 1758812 | 1760128 | aspartate kinase | *Enterococcus faecium* | e-113 |
|  |  |  |  |  |  |  |  |
| XVIII | 18 | 1415 | 1775962 | 1776174 | hypothetical protein | none |  |
|  |  | 1416 | 1776944 | 1777525 | hypothetical protein | none |  |
|  |  | 1417 | 1777450 | 1777355 | hypothetical protein | none |  |
|  |  | 1418 | 1778158 | 1777466 | possible transcriptional regulator | none |  |
|  |  | 1419 | 1778344 | 1778544 | hypothetical protein | none |  |
|  |  | 1420 | 1778754 | 1778825 | hypothetical protein | none |  |
|  |  | 1421 | 1778825 | 1779562 | hypothetical protein | none |  |
|  |  | 1422 | 1779581 | 1780033 | hypothetical protein | none |  |
|  |  | 1423 | 1780051 | 1780377 | hypothetical protein | none |  |
|  |  | 1424 | 1780374 | 1780478 | hypothetical protein | none |  |
|  |  | 1425 | 1780468 | 1780737 | hypothetical protein | none |  |
|  |  | 1426 | 1781048 | 1781344 | hypothetical protein | none |  |
|  |  | 1427 | 1781348 | 1781608 | hypothetical protein | none |  |
|  |  | 1428 | 1781598 | 1783643 | possible DNA replication protein | *Clostridium thermocellum* | e-60 |
|  |  | 1429 | 1784078 | 1784671 | hypothetical protein | none |  |
|  |  | 1430 | 1784702 | 1784866 | hypothetical protein | none |  |
|  |  | 1431 | 1784869 | 1785930 | probable integrase | *Clostridium perfringens* | e-45 |
|  |  | 1432 | 1785987 | 1786106 | hypothetical protein | none |  |
|  |  |  |  |  |  |  |  |
| XIX | 7 | 1609 | 1963649 | 1964059 | conserved hypothetical protein | *Mycoplasma penetrans* | e-40 |
|  |  | 1610 | 1964077 | 1964643 | possible integral membrane protein | *Streptococcus mutans* | e-30 |
|  |  | 1611 | 1964703 | 1964831 | conserved hypothetical protein | *Yersinia pestis* | e-8 |
|  |  | 1612 | 1964834 | 1965013 | hypothetical protein | none |  |
|  |  | 1613 | 1965023 | 1965472 | possible flavodoxin protein | *Archaeoglobus fulgidus* | e-8 |
|  |  | 1614 | 1965491 | 1966462 | possible esterase | *Mesorhizobium loti* | e-71 |
|  |  | 1615 | 1966463 | 1966519 | hypothetical protein | none |  |
|  |  |  |  |  |  |  |  |
| XX | 6 | 1617 | 1967186 | 1967737 | possible transcriptional regulator | *Oenococcus oeni* | e-13 |
|  |  | 1618 | 1967786 | 1967935 | conserved hypothetical protein | *Streptococcus agalactiae* | e-6 |
|  |  | 1619 | 1967978 | 1968121 | hypothetical protein | none |  |
|  |  | 1620 | 1968129 | 1968314 | hypothetical protein | none |  |
|  |  | 1621 | 1968583 | 1969827 | transposase | *Crocospaera watsonii* | e-102 |
|  |  | 1622 | 1969990 | 1970484 | conserved hypothetical protein | *Streptococcus pyogenes* | e-23 |
|  |  |  |  |  |  |  |  |
| XXI | 9 | 1662 | 2024189 | 2025301 | bacteriophage integrase | *Thermoanaerobacter tengcongensis* | e-33 |
|  |  | 1663 | 2025285 | 2025833 | hypothetical protein | none |  |
|  |  | 1664 | 2026091 | 2026192 | hypothetical protein | none |  |
|  |  | 1665 | 2026189 | 2026266 | hypothetical protein | none |  |
|  |  | 1666 | 2026263 | 2026469 | hypothetical protein | none |  |
|  |  | 1667 | 2026486 | 2027011 | pseudogene of prophage antirepressor | *Bacillus weihenstephanensis* | e-26 |
|  |  | 1668 | 2027004 | 2027339 | hypothetical protein | none |  |
|  |  | 1669 | 2027332 | 2027514 | hypothetical protein | none |  |
|  |  | 1670 | 2027558 | 2027881 | hypothetical protein | none |  |
|  |  |  |  |  |  |  |  |
| XXII | 10 | 1672 | 2029076 | 2030008 | possible bacteriophage endonuclease | *Bacillus halodurans* | e-7 |
|  |  | 1673 | 2030056 | 2030718 | possible bacteriophage antirepressor | *Clostridium tetani* | e-34 |
|  |  | 1674 | 2030756 | 2031412 | conserved hypothetical protein | *Desulfovibrio desulfuricans* | e-11 |
|  |  | 1675 | 2031597 | 2032013 | hypothetical protein |  |  |
|  |  | 1676 | 2026263 | 2033854 | possible DNA-directed DNA polymerase II | *Clostridium thermocellum* | e-76 |
|  |  | 1677 | 2033885 | 2035804 | probable ATPase | *Clostridium thermocellum* | e-26 |
|  |  | 1678 | 2036369 | 2036806 | probable bacteriophage protein | *Streptococcus mitis* | e-9 |
|  |  | 1679 | 2036807 | 2036989 | hypothetical protein | none |  |
|  |  | 1680 | 2037013 | 2037204 | hypothetical protein | none |  |
|  |  | 1681 | 2037206 | 2037310 | hypothetical protein | none |  |
|  |  |  |  |  |  |  |  |
| XXIII | 10 | 1683 | 2038001 | 2038411 | conserved hypothetical bacteriophage protein | *Clostridium perfringens* | e-10 |
|  |  | 1684 | 2038549 | 2039034 | probable bacteriophage terminase, small subunit | *Clostridium perfringens* | e-11 |
|  |  | 1685 | 2039035 | 2040759 | possible bacteriophage terminase, large subunit | *Clostridium perfringens* | e-77 |
|  |  | 1686 | 2040756 | 2041970 | probable bacteriophage portal protein | *Clostridium tetani* | e-91 |
|  |  | 1687 | 2041963 | 2042673 | ATP-dependent endopeptidase Clp | *Escherichia coli* | e-24 |
|  |  | 1688 | 2042678 | 2043784 | capsid protein | *Desulfitobacterium hafniense* | e-49 |
|  |  | 1689 | 2043796 | 2044089 | possible bacteriophage packaging protein | *Clostridium perfringens* | e-5 |
|  |  | 1690 | 2044082 | 2044417 | hypothetical protein | none |  |
|  |  | 1691 | 2044422 | 2044841 | hypothetical protein | none |  |
|  |  | 1692 | 2044841 | 2045281 | hypothetical protein | none |  |
|  |  |  |  |  |  |  |  |
| XXIV | 10 | 1694 | 2046372 | 2046803 | bacteriophage PBSX protein XkdM | *Clostridium tetani* | e-24 |
|  |  | 1695 | 2046817 | 2047206 | hypothetical protein | none |  |
|  |  | 1696 | 2047209 | 2047355 | hypothetical protein | none |  |
|  |  | 1697 | 2047426 | 2047671 | hypothetical protein | none |  |
|  |  | 1698 | 2047745 | 2050096 | conserved hypothetical bacteriophage protein | *Bifidobacterium longum* | e-11 |
|  |  | 1699 | 2050111 | 2050656 | hypothetical protein | none |  |
|  |  | 1700 | 2050666 | 2051646 | phage-like element PBSX family protein XkdQ | *Clostridium tetani* | e-10 |
|  |  | 1701 | 2051639 | 2052163 | hypothetical protein | none |  |
|  |  | 1702 | 2052142 | 2052594 | hypothetical protein | none |  |
|  |  | 1703 | 2052591 | 2053649 | bacteriophage PBSX protein XkdT | *Clostridium tetani* | e-63 |
|  |  |  |  |  |  |  |  |
| XXV | 26 | 1820 | 2174023 | 2174136 | hypothetical protein | none |  |
|  |  | 1821 | 2174309 | 2175025 | hypothetical protein | none |  |
|  |  | 1822 | 2175029 | 2176399 | hypothetical protein | none |  |
|  |  | 1823 | 2176396 | 2176710 | hypothetical protein | none |  |
|  |  | 1824 | 2176779 | 2177429 | hypothetical protein | none |  |
|  |  | 1825 | 2177447 | 2177743 | hypothetical protein | none |  |
|  |  | 1826 | 2177512 | 2177793 | hypothetical protein | none |  |
|  |  | 1827 | 2177756 | 2177887 | hypothetical protein | none |  |
|  |  | 1828 | 2177884 | 2178345 | conserved hypothetical protein | *Staphylococcus aureus* | e-7 |
|  |  | 1829 | 2178338 | 2178697 | hypothetical protein | none |  |
|  |  | 1830 | 2178758 | 2179150 | hypothetical protein | none |  |
|  |  | 1831 | 2179165 | 2179311 | hypothetical protein | none |  |
|  |  | 1832 | 2179370 | 2179567 | hypothetical protein | none |  |
|  |  | 1833 | 2179654 | 2181795 | topoisomerase | *E. coli* | e-103 |
|  |  | 1834 | 2181810 | 2184578 | conserved hypothetical protein | *Streptococcus thermophilus* | e-26 |
|  |  | 1835 | 2184869 | 2184967 | hypothetical protein | none |  |
|  |  | 1836 | 2185082 | 2186038 | hypothetical protein | none |  |
|  |  | 1837 | 2186094 | 2186888 | hypothetical protein | none |  |
|  |  | 1838 | 2186904 | 2187209 | hypothetical protein | none |  |
|  |  | 1839 | 2188971 | 2189510 | possible protease TraF | *Prosthecochloris aestuarii* | e-5 |
|  |  | 1840 | 2189667 | 2189810 | hypothetical protein | none |  |
|  |  | 1841 | 2189829 | 2190401 | hypothetical protein | none |  |
|  |  | 1842 | 2190438 | 2190794 | hypothetical protein | none |  |
|  |  | 1843 | 2190865 | 2191131 | hypothetical protein | none |  |
|  |  | 1844 | 2191143 | 2192234 | possible zeta toxin | *Streptococcus pyogenes* | e-45 |
|  |  | 1845 | 2192352 | 2192513 | hypothetical protein |  |  |
|  |  |  |  |  |  |  |  |
| XXVI | 17 | 1847 | 2193729 | 2194400 | hypothetical protein | none |  |
|  |  | 1848 | 2194543 | 2194635 | hypothetical protein | none |  |
|  |  | 1849 | 2194721 | 2195722 | hypothetical protein | none |  |
|  |  | 1850 | 2195750 | 2196142 | hypothetical protein | none |  |
|  |  | 1851 | 2196255 | 2196350 | hypothetical protein | none |  |
|  |  | 1852 | 2196491 | 2197396 | possible phage resistance protein | *Lactococcus lactis* | e-6 |
|  |  | 1853 | 2197456 | 2197746 | hypothetical protein | none |  |
|  |  | 1854 | 2197746 | 2198333 | conserved hypothetical protein | *Treponema denticola* | e-5 |
|  |  | 1855 | 2198484 | 2199341 | hypothetical protein | *none* |  |
|  |  | 1856 | 2199357 | 2199770 | hypothetical protein | *none* |  |
|  |  | 1857 | 2199754 | 2200212 | hypothetical protein | *none* |  |
|  |  | 1858 | 2200263 | 2200868 | possible DNA integrase | *Shewanella oneidensis* | e-19 |
|  |  | 1859 | 2201264 | 2203675 | pseudogene of probable plasmid mobilization protein | *Actinobacillus pleuropneumoniae* | e-32 |
|  |  | 1860 | 2203663 | 2204184 | hypothetical protein | none |  |
|  |  | 1861 | 2204174 | 2204473 | hypothetical protein | none |  |
|  |  | 1862 | 2204508 | 2204825 | hypothetical protein | none |  |
|  |  | 1863 | 2204934 | 2205188 | hypothetical protein | none |  |
|  |  |  |  |  |  |  |  |
| XXVII | 12 | 1865 | 2205860 | 2206081 | hypothetical protein | none |  |
|  |  | 1866 | 2206078 | 2206322 | pseudogene of possible plasmid addiction protein | none |  |
|  |  | 1867 | 2206485 | 2206805 | hypothetical protein | none |  |
|  |  | 1868 | 2206819 | 2207523 | probable conjugal transfer protein TrbF | *Caulobacter crescentus* | e-17 |
|  |  | 1869 | 2207535 | 2208371 | probable conjugal transfer protein TrbG | *Caulobacter crescentus* | e-24 |
|  |  | 1870 | 2208381 | 2209583 | probable conjugal transfer protein TrbI | *Mesorhizobium loti* | e-45 |
|  |  | 1871 | 2209586 | 2211616 | probable conjugal transfer protein TraG | *Mesorhizobium loti* | e-67 |
|  |  | 1872 | 2211762 | 2212076 | hypothetical protein | none |  |
|  |  | 1873 | 2212045 | 2213010 | possible type II/IV secretion system protein | *Rhizobium sp.* | e-67 |
|  |  | 1874 | 2213019 | 2213282 | hypothetical protein | none |  |
|  |  | 1875 | 2213293 | 2216031 | possible type IV secretion system protein VirB4 | *Mesorhizobium loti* | e-145 |
|  |  | 1876 | 2216069 | 2216251 | hypothetical protein | none |  |
|  |  | 1877 | 2216275 | 2217012 | possible outer membrane protein | *Pasteurella multocida* | e-18 |
|  |  | 1878 | 2217119 | 2217268 | hypothetical protein | none |  |
|  |  | 1879 | 2217243 | 2218775 | possible cassette chromosome recombinase | *Moorella thermoacetica* | e-82 |
|  |  |  |  |  |  |  |  |
| XXVIII | 5 | 1949 | 2298529 | 2298287 | probable membrane protein | *Brucella melitensis* | e-7 |
|  |  | 1950 | 2299052 | 2298558 | hypothetical protein | none |  |
|  |  | 1951 | 2299473 | 2299078 | hypothetical protein | none |  |
|  |  | 1952 | 2300585 | 2299671 | branched-chain-amino-acid transaminase | *Methanothermo-bacter thermautotrophicus* | e-103 |
|  |  | 1953 | 2300703 | 2301086 | conserved hypothetical protein | none |  |
